# Susceptibility Source Separation Unveils Paramagnetic and Diamagnetic Trajectories in Healthy Brains From 5 to 90 Years

**DOI:** 10.1002/nbm.70349

**Published:** 2026-07-06

**Authors:** Tereza Beatriz Oliveira Assunção, Nashwan Naji, Peter Seres, Christian Beaulieu, Alan H. Wilman

**Affiliations:** ^1^ Department of Biomedical Engineering University of Alberta Edmonton Alberta Canada; ^2^ Department of Radiology and Diagnostic Imaging University of Alberta Edmonton Alberta Canada

**Keywords:** healthy lifespan, human brain, source separation, susceptibility

## Abstract

Susceptibility source separation (SSS) enables independent evaluation of both paramagnetic iron and diamagnetic myelin in the human brain. The aim of this work was to analyze healthy brain lifespan trajectories of paramagnetic and diamagnetic susceptibilities in deep gray matter (DGM) and white matter (WM) from a large database (339 subjects, 5 to 90 years), all acquired at 3 T on the same scanner, using χ‐separation and comparing it to two other common SSS methods (χ‐sepnet and APART‐QSM). A 3D multiple echo gradient echo sequence was used to measure phase, R2* and QSM, as well as dual echo fast spin echo to measure R2. The mean value of WM and DGM regions was calculated for SSS output maps and plotted against age to evaluate trajectories across the lifespan. With χ‐separation, most DGM regions showed an increasing trend with age in the paramagnetic map, except for thalamus, which showed an inverted‐U quadratic trajectory, and all DGM regions showed an increase in diamagnetic content with age. In WM, for the paramagnetic maps, body of corpus callosum, splenium and corticospinal tract showed an inverted quadratic trajectory, cingulum an increasing exponential, while no significant changes were seen in genu. For the diamagnetic WM maps, all regions followed a similar trajectory, increase in early life, peak around 40 to 60 years, and decrease with age. The different SSS approaches yielded different curve shapes and mean values in many instances. For example, APART‐QSM had consistently lower ppb values, χ‐sepnet had biologically unexpected results for early ages, and χ‐separation data had higher standard deviation of best‐fit residuals when compared to the other evaluated methods for all analyzed regions and maps. All SSS methods enabled depiction of independent iron and myelin trends; however, different results between methods suggest caution in choosing SSS methods and the need for further methodological advances.

## Graphical abstract

This work analyzed healthy brain lifespan trajectories of paramagnetic iron and diamagnetic myelin with three common susceptibility source separation methods (χ‐separation, χ‐sepnet and APART‐QSM). Deep gray and white matter regions of 339 subjects (5–90 years) were studied. All methods enabled depiction of independent paramagnetic and diamagnetic trends, with substantial variations between methods.

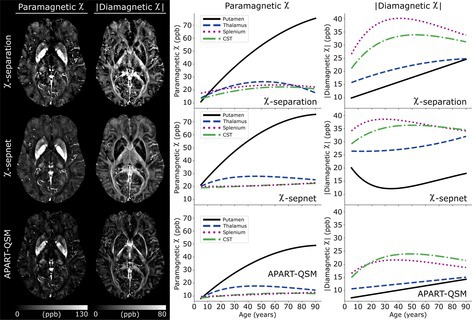

**Abbreviations**
AIC: Akaike information criterion
APART‐QSM: iterAtive magnetic suscePtibility sources separation
CC: corpus callosum
CST: corticospinal tract
DGM: deep gray matter
D_r_
: relaxometric constant
FDR: false discovery rate
FSL: FMRIB software library
MEDI: morphology‐enabled dipole inversion
MEGE: multi‐echo gradient echo
MPRAGE: magnetization‐prepared rapid gradient echo
MWF: myelin water fraction
ppb: part per billion
ppm: part per million
QSM: quantitative susceptibility mapping
R: pearson correlation coefficients
R2: irreversible transverse relaxation rate
R2*: effective transverse relaxation rate
R2′: reversible transverse relaxation rate
RF: radio frequency
ROI: regions‐of‐interest
ROMEO: Rapid Opensource Minimum spanning treE algorithm
SSS: susceptibility source separation
STAR: STreaking Artifact Reduction
TSE: turbo spin echo
V‐SHARP: variable‐radius sophisticated harmonic artifact reduction for phase
WM: white matter

## 1 Introduction

Paramagnetic iron and diamagnetic myelin are the two main susceptibility sources in the human brain [1]. Both are important for brain function as iron is required for myelin synthesis and oxygen transportation, whereas myelin acts as the insulating sheath around axons, enabling saltatory conduction. Both quantities are expected to vary across the lifespan in healthy subjects, for example, myelin in White Matter (WM), as measured from in vivo myelin water imaging, typically increases until a peak in mid‐life followed by a decrease with age [2, 3], similarly, iron concentrations in Deep Gray Matter (DGM), as measured from postmortem chemistry, increase in early ages, plateau in middle age, but then increase with further aging in many regions [4, 5]. Different patterns can be seen in neurological diseases such as in multiple sclerosis, where demyelination and possibly remyelination occurs while iron accumulation is seen in the basal ganglia and in active lesions [6, 7]. More generally, brain iron accumulation is observed in select regions in many neurodegenerative diseases including amyotrophic lateral sclerosis, dementia and Parkinson's disease [8, 9, 10]. Hence, understanding normal levels of iron and myelin across the brain is important for interpreting changes in disease.

To image these sources in the brain, different MRI techniques have been used, including myelin water fraction (MWF) [11, 12] and magnetization transfer [13, 14] for myelin, and transverse relaxation [15, 16] and quantitative susceptibility mapping (QSM) [17, 18], for iron. A downside of most of these methods is that they quantify either iron or myelin, but not both at the same time. Focusing on QSM, it is sensitive to both paramagnetic iron and diamagnetic myelin, and even though most commonly used for imaging iron, it has been used for measuring demyelination as well [19]. However, the effects of paramagnetic and diamagnetic sources cannot be distinguished, as QSM provides a net susceptibility map. Hence, if both sources coexist in the same voxel, they can partly cancel each other out. Additional information from an R2* map, alongside QSM, has been used to analyze iron and myelin simultaneously [20, 21, 22], as R2* and QSM have similar directional effects for iron but opposing effects for myelin. However, these works typically only identified the dominant component in each voxel, as it is difficult to quantify the individual iron and myelin components.

An important step forward was to apply the relationship between susceptibility and the reversible transverse relaxation rate R2′ to separate the diamagnetic and paramagnetic components via a process called susceptibility source separation (SSS). This method, which was first proposed in 2021 by Shin et al. [23], generates independent paramagnetic and diamagnetic susceptibility maps, overcoming the net susceptibility issue of QSM and enabling the evaluation of both iron and myelin. To date, different SSS methods have been proposed [24, 25, 26, 27, 28]. These methods use different computation and base models. The original method, named χ‐separation [23], uses an iterative solver, whereas χ‐sepnet [24] uses deep‐learning to predict paramagnetic and diamagnetic maps. APART‐QSM [25] also uses an iterative approach, however, it calculates the relaxometric constant (D_r_) for each voxel, in contrast to χ‐separation, which has a fixed D_r_ for the whole brain. Three previous studies utilized SSS methods to analyze susceptibility component trajectories with age in healthy cohorts. Min et al. [29] utilized χ‐sepnet‐R2* [24] data from 106 adults (aged from 27 to 85 years). Lao et al. [30] used APART‐QSM [25] in 2 cohorts (cohort 1: 494 subjects, 18–80 years; and cohort 3: 32 subjects, 4–39 years; acquired on different scanners). For the larger adult‐only cohort, R2′ was approximated from R2* (using cohort 2: 13 young adults), while for the smaller one, R2′ was calculated by subtracting R2 from R2*. Zhou et al. [31] utilized χ‐sepnet‐R2* [24] data from 131 healthy adults (aged from 21 to 89 years). From these three studies, findings within DGM regions‐of‐interest (ROIs) showed linear increases with aging for the paramagnetic map in caudate and putamen, linear decrease in thalamus, and each study showed a different pattern for globus pallidus, including an exponential increase [30], a linear decrease [31] and no significant changes [29] in paramagnetic content with aging. As for the diamagnetic content within DGM ROIs, most of these ROIs showed a linear increase in diamagnetic content across the lifespan. Regarding WM ROIs, within the paramagnetic map, genu had no significant changes with age, body of corpus callosum (CC) showed either a linear decreasing trend [29] or no changes with age [31], while splenium showed linear increasing trends in two [30, 31] studies and no changes with age in another [29]. As for the diamagnetic content within WM ROIs, splenium showed a significant trajectory in all studies; two studies [29, 31] found a decrease in diamagnetic content, while the other [30] found an inverted U‐shape trajectory with a peak at 39 years old. Min et al. [29] found no significant changes for both genu and body of CC diamagnetic content; while Zhou et al. [31] found a linear decreasing trend and an inverted U‐shape for these ROIs, respectively. The inclusion of a limited number of children (< 20 subjects younger than 15 years, cohort 3 [30]) resulted in no clear changes when comparing the young cohort trajectories to the adult‐only lifespan trends; except in globus pallidus, which showed a steep increase for both susceptibility sources from 4 to 20 years, that was not seen in the older cohort.

However, none of these studies showed data from young to elderly ages using a single scanner protocol. Furthermore, past lifespan studies with SSS have only utilized one technique in each study; hence, comparisons between techniques have not been evaluated within the same lifespan cohort. Thus, the aim of this work was to analyze lifespan trajectories using χ‐separation in a large database ranging from 5 to 90 years old, and evaluate the benefits of SSS over net susceptibility mapping. Additionally, two other SSS methods (χ‐sepnet and APART‐QSM) were studied to compare how different methods may impact age trajectories of paramagnetic and diamagnetic susceptibility.

## 2 Methods

### 2.1 Subjects

Three hundred and thirty‐nine healthy subjects from 5 to 90 years old (194 females, 36 ± 21 years; 145 males, 38 ± 22 years), who were previously part of a larger study [32], were scanned at 3 T (Siemens Prisma, Erlangen, Germany) after providing informed consent. The original database had data from 378 subjects, however 28 subjects were excluded for not having all of the needed sequences and an additional 11 subjects for low image quality. From the 339 subjects included in this study, their median income was $101.000–150.000 CAD per year and 68% identified themselves as Caucasian. More detailed demographic data can be found in Treit et al. [32]. Results for R2* and QSM have been previously reported [33] in a study that combined this dataset with an additional 159 subjects from another study.

### 2.2 MRI Protocol

A 3D Multi‐Echo Gradient Echo (TR 37 ms, TE1 3.82 ms with echo spacing 5.49 ms, 6 echoes, flip angle 13°, FoV 240 × 203 × 150 mm^3^, 0.9 × 0.9 × 1.7 mm^3^ spatial resolution, scan time 5 min 30 s) was used for R2* mapping, phase and QSM. Brain segmentation utilized 3D MPRAGE (TR 1800 ms, TE 2.37 ms, TI 900 ms, flip angle 8°, FoV 250 × 250 × 177 mm^3^, 0.9 × 0.9 × 0.9 mm^3^ spatial resolution, scan time 3 min 39 s). For the R2 map acquisition, a 2D dual echo Turbo Spin Echo (TR 4000 ms, TE1 10 ms, TE2 93 ms, turbo factor 8, 160° refocusing, FoV 240 × 180 × 143.5 mm^3^, 0.9 × 0.9 × 3.5 mm^3^ spatial resolution, scan time 2 min 02 s) was used along with a B_1_^+^ mapping sequence [34] (1.3 × 1.3 × 3.0 mm^3^ spatial resolution, scan time 39 s).

### 2.3 Regions of Interest Segmentation

Four representative deep gray matter (DGM) structures were chosen, including caudate, globus pallidus, putamen, and thalamus. Five white matter (WM) ROIs were selected with different tract orientations, including splenium, body, and genu of corpus callosum (CC) (transverse orientation), corticospinal tract (CST) (craniocaudal orientation), and cingulum (anteroposterior orientation). DGM structures were segmented in MPRAGE space using volBrain [35], and WM ROIs were segmented by registering the Johns Hopkins University White‐matter Atlas [36] to MPRAGE space in FSL [37]. Then, all ROIs were registered to Multi‐Echo Gradient Echo (MEGE) space with FSL [37].

### 2.4 Relaxation Maps Reconstruction (R2, R2* and R2′)

R2 maps were reconstructed by Bloch modeling the dual‐echo Turbo Spin Echo (TSE) decay with measured flip angles, as performed by McPhee and Wilman [38]. This method consists of dictionary fitting, modeled by Bloch equations, based on the exact pulse sequence RF and gradient waveforms, and accounting for B_1_^+^ variations. R2* maps were calculated by solving the mono‐exponential decay model of the MEGE magnitude images as a nonlinear minimization [39]. R2′ maps were calculated by subtracting R2 from R2* after interpolation of R2 maps to the same R2* resolution and registering R2 maps to MEGE space using FSL. Negative R2′ values, often found in CSF, were set to zero since such values are not realistic.

### 2.5 Susceptibility Source Separation Processing

To perform SSS, three different methods were used: χ‐separation [23], χ‐sepnet [24] and APART‐QSM [25]. χ‐separation, which was the first SSS method proposed, is an iterative method that links R2′ to the total susceptibility by a relaxometric constant (D_r_), which is the slope of the linear regression between R2′ and QSM. χ‐sepnet is a deep learning method, more specifically, a 3D U‐net trained with labels from χ‐separation using multi‐orientation head data. For both methods, a single default D_r_ is used for the whole brain. APART‐QSM is also an iterative method, modeled similarly to χ‐separation, however, the D_r_ is calculated iteratively for each voxel. More details on each method can be found in their literature [23, 24, 25]. The reconstruction codes for each method were downloaded from their respective GitHub repositories, as follows: χ‐separation (https://github.com/SNU‐LIST/chi‐separation, version 1.1.3, accessed on February 2025); χ‐sepnet (https://github.com/SNU‐LIST/chi_sepnet, version 1.1.3, accessed on February 2025); and APART‐QSM (https://github.com/AMRI‐Lab/APART‐QSM, accessed on June 2023).

The inputs for SSS have similarities between methods; all of them require data from a MEGE scan and accept either an R2 or R2′ map. More specifically, for χ‐sepnet, the inputs include: R2′ map, tissue field map, brain mask, imaging frequency and B_0_ direction. χ‐separation requires the same inputs, with the addition of magnitude images, noise maps and QSM for an initial guess of the total susceptibility. The inputs for APART‐QSM include: R2 map, tissue field map, magnitude images, brain mask, and parameters of B_0_ field and direction and TEs. However, these methods differ in the D_r_ input; for χ‐sepnet, the standard value is 114 Hz/ppm for both paramagnetic and diamagnetic components, for χ‐separation it is 137 Hz/ppm, and for APART‐QSM the initial guess for D_r_ is set to 323.5 Hz/ppm. These standard D_r_ values for χ‐sepnet and χ‐separation were calculated, in their respective original studies [23, 24], by a linear regression between QSM and R2′ values of a few subjects using iron‐rich DGM ROIs, while APART‐QSM uses a theoretical value from Yablonskiy and Haacke [40] for the initial guess D_r_, which is then adjusted iteratively for each voxel, while solving for the paramagnetic and diamagnetic maps separation.

### 2.6 Quantitative Susceptiblity Mapping Processing

Two of the three SSS methods used in this work can take QSM for initializing the total susceptibility. However, they differ on the recommended methodology for the dipole inversion step. χ‐separation uses QSM calculated with the MEDI [41] algorithm, while APART‐QSM recommends the STAR [42] algorithm. Because of this distinction and to compare the influence of the QSM methodology in the source separation output, both QSM algorithms were performed and used as inputs for each of the SSS methods. The main results are only shown for χ‐separation with MEDI and APART‐QSM with STAR, as these are the recommendations of the original references, and are referred to as χ‐separation and APART‐QSM, respectively. The results for χ‐separation with STAR and APART‐QSM with MEDI are referred to as χ‐separation_STAR and APART‐QSM_MEDI, respectively.

Regardless of the algorithm used for the dipole inversion, the phase volumes of all echoes were first unwrapped with phase offset removal using ROMEO [43], then normalized by the imaging frequency and TE to obtain field maps, and combined using T2*‐weighted average when the SSS method requires a single field map. Background field contributions were removed using V‐SHARP [44] with a 12 mm kernel radius.

### 2.7 Data Analysis

A qualitative assessment was done by visually analyzing paramagnetic susceptibility ($\chi_{\text{para}}$) and absolute diamagnetic susceptibility ($\left|\chi_{\mathrm{dia}}\right|$) maps of five representative subjects across the age range from 5 to 80 years. For quantitative assessment, $\chi_{\text{para}}$ and $\left|\chi_{\mathrm{dia}}\right|$ were calculated within the aforementioned (in Section 2.3) DGM and WM segmented ROIs for all 339 subjects, and plotted against age to evaluate trajectories across the lifespan. Linear, quadratic, cubic, exponential and Poisson fits were tested, and the best fit was chosen based on the smallest Akaike information criterion (AIC) value. *p*‐Value and R^2^ were also calculated for each fit. Similar data from relaxation (R2′ and R2*) and QSM maps were also calculated. Differences between left vs. right sides were tested with lateralization index, with a|mean index| > 0.2 [45] being considered as significantly different. For the other statistical analysis done in this study, significant differences were considered for any α < 0.05.

To evaluate sex differences, a permutation‐based test was used; males and females were fitted separately, using the same aforementioned approach for the choice of the best fit. Then, males and female labels were randomly permuted within subjects and the permuted data was fitted (males and females separately with the original best fit curve). This permutation was performed 10,000 times and, for each iteration, as well as for the original data, the mean absolute difference between the predicted values from 5 to 90 years was calculated. Then, a *p*‐value was evaluated as the proportion of the iterations that resulted in a mean absolute difference equal to or higher than the one found with the original data. *p*‐Values within each SSS method (18 comparisons per method, from two susceptibility maps and nine ROIs) were FDR corrected; the α, after correction, was used for considering significant differences.

Linear correlations between the results of the three different SSS methods were evaluated. Pearson correlation coefficients (R) were calculated for each segmented ROI with $\chi_{\text{para}}$ and $\left|\chi_{\mathrm{dia}}\right|$; three coefficients were acquired for each case, as the methods were paired for comparison.

### 2.8 Influence of Relaxometric Constant

To evaluate the influence of D_r_ in $\chi_{\text{para}}$ and $\left|\chi_{\mathrm{dia}}\right|$, the 3 evaluated SSS methods were applied to a single example subject (male, 40 years old) with different D_r_ values, ranging from 0.0001 to 350 Hz/ppm in 5 Hz/ppm steps, in addition to the three standard values used for each SSS method (114, 137 and 323.5 Hz/ppm). The mean values within all nine aforementioned ROIs for all produced maps were calculated and plotted against the range of D_r_s used as input. Mean ppb values, in all SSS output maps and ROIs, decreased hyperbolically with increasing D_r_ values (data not shown). Hyperbolical fitting (following the model χ = *a* + *b*/D_r_) of this data was done for one DGM ROI (Caudate) and one WM (Splenium). Furthermore, five D_r_ values (50, 114, 137, 230 and 323.5 Hz/ppm) were chosen to be used in a lifespan trajectories analysis, for two example ROIs (Caudate and Splenium). SSS maps with these 5 D_r_ values were processed for the whole cohort, and mean values within these 2 ROIs were calculated and best fit curves determined.

## 3 Results

### 3.1 Qualitative Assessment

**FIGURE 1:**
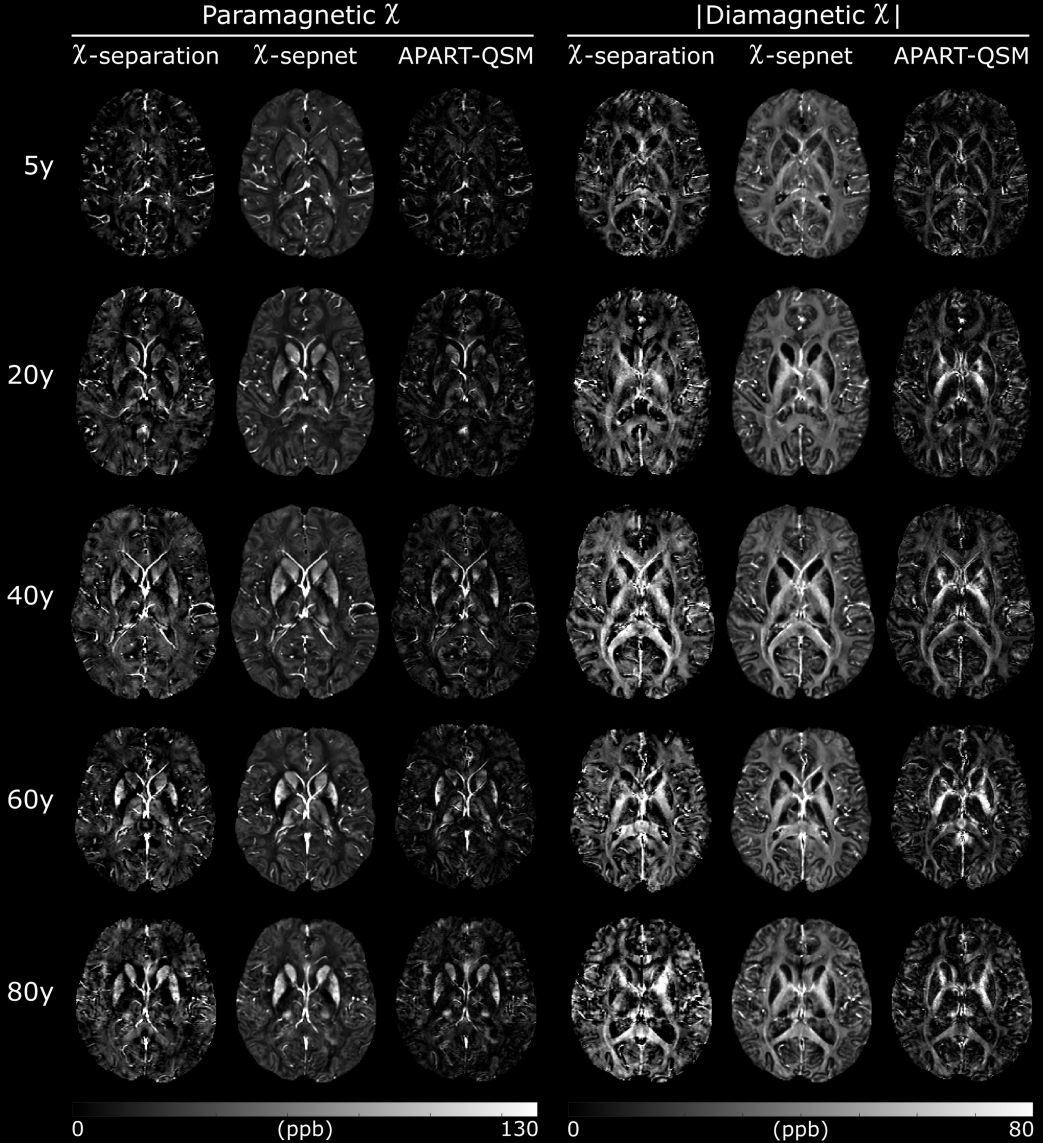
χ‐separation paramagnetic χ and absolute diamagnetic χ maps from 5 representative subjects across the lifespan. The maps illustrate clear distinction between paramagnetic iron and diamagnetic myelin. Paramagnetic content in the deep gray matter increases with age, reflective of iron increases. Increased diamagnetic content in white matter from early age to young adulthood is reflective of increasing myelination.

Example $\chi_{\text{para}}$ and $\left|\chi_{\mathrm{dia}}\right|$ maps from across the lifespan are shown in Figure 1 for central brain regions with each SSS method. The distinct separation of paramagnetic and diamagnetic components was evident in all methods, but there was strikingly different contrast between methods. Most obvious was the much lower ppb values with APART‐QSM in comparison to χ‐separation and the much smoother maps with Χ‐sepnet. General trends for all SSS methods included an increase in paramagnetic content in DGM with age. Example relaxation maps and QSM, for the same subjects included in Figure 1, are shown in Figure S1.

### 3.2 Hemispheric Asymmetry and Sex Differences

No significant differences were found between left and right hemispheres (males and females together), thus, both sides were averaged for all measures. Differences between sexes (with left and right averaged) were found, with χ‐separation, for thalamus, with both $\chi_{\text{para}}$ and $\left|\chi_{\mathrm{dia}}\right|$, and for caudate $\left|\chi_{\mathrm{dia}}\right|$. Males showed increased diamagnetic content in caudate throughout the whole lifespan, with a mean difference between males and females of 2.5 ppb; while in the thalamus, females showed higher concentrations of both susceptibility sources (mean difference of 0.7 ppb for both) in early life, males had higher values in mid‐life (from 13 to 60 years old and 1 ppb mean difference for $\left|\chi_{\mathrm{dia}}\right|$, and from 20 to 75 years and 3 ppb mean difference for $\chi_{\text{para}}$), followed by higher female values (3 and 3.6 ppb mean difference for $\left|\chi_{\mathrm{dia}}\right|$ and $\chi_{\text{para}}$, respectively) with aging. Plots for these cases, with males and females fitted separately, can be found in Figure S2. These same ROIs and maps also showed significant sex differences with χ‐sepnet and APART‐QSM (data not shown). However, as most of the comparisons did not show any significant sex differences, for ease of comparison, the following results will be shown with males and females fitted together.

### 3.3 Lifespan Trajectories With χ‐Separation

**FIGURE 2:**
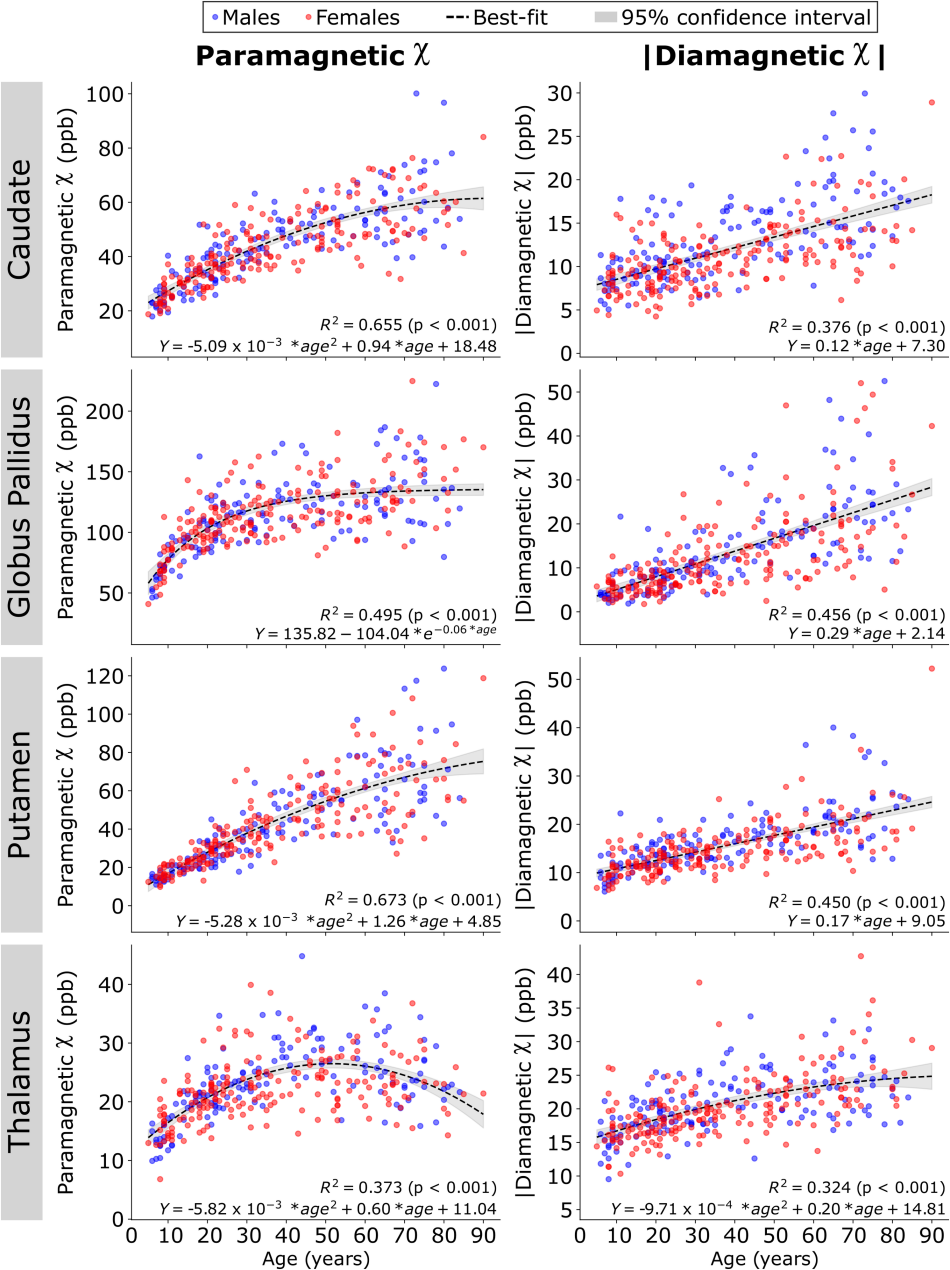
Data points and best‐fit curves, within deep gray matter regions, for χ‐separation $\chi_{\text{para}}$ and $\left|\chi_{\mathrm{dia}}\right|$ maps. Left and right hemispheres were averaged for each region. Males (blue) and females (red) were combined for the curve fitting.

All best fits were significant (*p* < 0.05) for χ‐separation, with default initial QSM, except for $\chi_{\text{para}}$ genu. Regarding the DGM regions, in $\chi_{\text{para}}$, caudate and putamen both best fit a quadratic trajectory showing an increase in paramagnetic content from 5 to 90 years old. Globus pallidus best fits an exponential, increasing in early ages and reaching a plateau at 44 years, with 58% of the change in ppb being reached by 20 years old. Thalamus best fits a quadratic, with paramagnetic content increasing in early life, reaching a peak at 52 years (with 54% of these change being reached by 20 years old), and decreasing later in life (Figure 2, Table 1). As for $\left|\chi_{\mathrm{dia}}\right|$, all DGM ROIs showed an increase across the lifespan, and most of them best fit an increasing linear trajectory, with the exception of thalamus, which best fits a quadratic (Figure 2, Table 1). All best fits and respective parameters for $\chi_{\text{para}}$ and $\left|\chi_{\mathrm{dia}}\right|$ for DGM regions can be found in Table 2.

**TABLE 1:** Values predicted by curves best fits for paramagnetic χ and absolute diamagnetic χ maps within deep gray matter regions.

		Value at 5 years (ppb)	Age at peak value (years)	Value at 90 years (ppb)	Age at 90% of total change from 5 years to peak (years)^a^	% change from 5 to 20 years (%)
Paramagnetic χ	Caudate					
	χ‐separation	23.0		61.4	64	31.6
	χ‐sepnet	30.1		66.6	60	42.7
	APART‐QSM	17.3		40.1	64	39.1
	Globus pallidus					
	χ‐separation	58.0		135.3	44	58.6
	χ‐sepnet	77.3		135.6	24	83.2
	APART‐QSM	43.7		85.4	17	93.8
	Putamen					
	χ‐separation	11.0		75.3	74	26.3
	χ‐sepnet	21.4		75.8	67	30.4
	APART‐QSM	8.1		49.0	66	30.7
	Thalamus					
	χ‐separation	13.9	52	17.9	37	54.1
	χ‐sepnet	20.3	45	25.3	30	68.8
	APART‐QSM	8.5	46	14.5	30	68.4
|Diamagnetic χ|	Caudate					
	χ‐separation	7.9		18.3	81	17.6
	χ‐sepnet	16.3	29	16.5	20	90.3
	APART‐QSM	4.7		11.1	81	17.6
	Globus pallidus					
	χ‐separation	3.6		28.4	81	17.6
	χ‐sepnet	6.4	22	17.0	17	98.4
	APART‐QSM	9.0	27	13.7	20	89.2
	Putamen					
	χ‐separation	9.9		24.6	81	17.6
	χ‐sepnet	20.1	34	18.0	23	82.4
	APART‐QSM	7.3		14.3	81	17.6
	Thalamus					
	χ‐separation	15.8		24.8	69	28.9
	χ‐sepnet^b^	26.4	14	32.0	11	
	APART‐QSM	10.7		15.1	81	17.6

^a^ For fits that do not have a peak, 90% of total change was calculated between 5 to 90 years old.

^b^ For thalamus absolute diamagnetic χ best fit with χ‐separation, the trajectory reached a peak at an age younger than 20 years old.

**TABLE 2:** Parameter values for curves best fits for paramagnetic χ and absolute diamagnetic χ maps within deep gray matter regions.

		Fit	A	B	C	D	E
Paramagnetic χ	Caudate						
	χ‐separation	Quadratic	18.48	0.94	−5.09E−03		
	χ‐sepnet	Exponential	68.61			−45.80	−0.03
	APART‐QSM	Exponential	42.10			−28.82	−0.03
	Globus pallidus						
	χ‐separation	Exponential	135.82			−104.04	−0.06
	χ‐sepnet	Exponential	135.65			−105.77	−0.12
	APART‐QSM	Exponential	85.43			−105.50	−0.19
	Putamen						
	χ‐separation	Quadratic	4.85	1.26	−5.28E−03		
	χ‐sepnet	Quadratic	15.21	1.27	−6.59E−03		
	APART‐QSM	Quadratic	3.38	0.96	−5.07E−03		
	Thalamus						
	χ‐separation	Quadratic	11.04	0.60	−5.82E−03		
	χ‐sepnet	Poisson	17.50			0.63	−0.02
	APART‐QSM	Poisson	5.15			0.75	−0.02
|Diamagnetic χ|	Caudate						
	χ‐separation	Linear	7.30	0.12			
	χ‐sepnet	Poisson	20.35			−0.97	−0.03
	APART‐QSM	Linear	4.33	0.08			
	Globus pallidus						
	χ‐separation	Linear	2.14	0.29			
	χ‐sepnet	Quadratic	6.89	−0.11	2.45E−03		
	APART‐QSM	Quadratic	9.38	−0.07	1.36E−03		
	Putamen						
	χ‐separation	Linear	9.05	0.17			
	χ‐sepnet	Poisson	24.18			−0.95	−0.03
	APART‐QSM	Linear	6.87	0.08			
	Thalamus						
	χ‐separation	Quadratic	14.81	0.20	−9.71E−04		
	χ‐sepnet	Quadratic	26.55	−0.03	9.72E−04		
	APART‐QSM	Linear	10.43	0.05			

*Note:* Fits were modeled based on the A–E parameters as follows: Linear = A+B·age; Quadratic = $A+B\cdot \mathrm{age}+C\cdot {\mathrm{age}}^{2}$; Exponential = $A+D\cdot e^{E\cdot \mathrm{age}}$; Poisson = $A+D\cdot \mathrm{age}\cdot e^{E\cdot \mathrm{age}}$.

**FIGURE 3:**
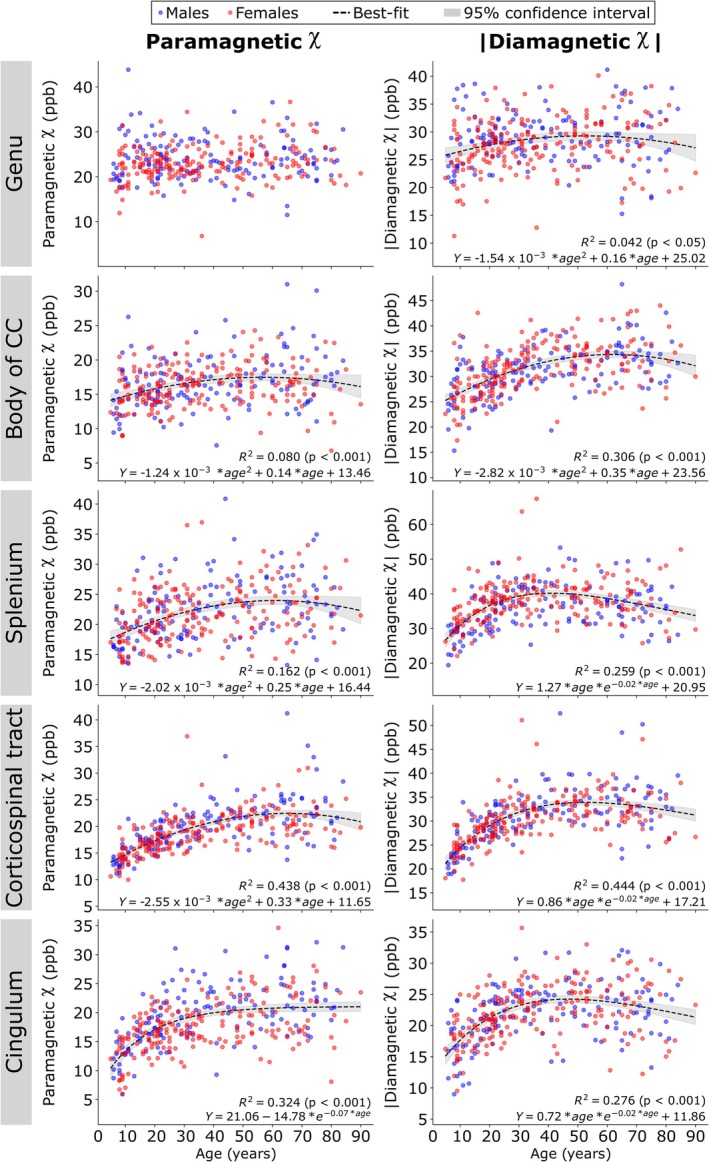
Data points and best‐fit curves, within white matter regions, for χ‐separation $\chi_{\text{para}}$ and $\left|\chi_{\mathrm{dia}}\right|$ maps. Left and right hemispheres were averaged for each region. Males (blue) and females (red) were combined for the curve fitting. Genu paramagnetic χ best fit was not significant, so no curve shown. CC, Corpus callosum.

In WM ROIs for $\chi_{\text{para}}$: genu did not change across the lifespan; cingulum best fits an exponential, showing an increase in early ages and reaching a plateau by 40 years old (with 62% of change being reached by 20 years old); while body, splenium and CST all best fit a quadratic trajectory, showing an increase in paramagnetic content in early life, reaching a peak around 60 years old and then decreasing with age (Figure 3, Table 3). For $\left|\chi_{\mathrm{dia}}\right|$, all WM ROIs showed a similar trend with increase in early ages and a decrease with age after a peak. More specifically, the body of CC best fits a quadratic, peaking at 62 years old. Splenium best fits a Poisson, with peak at 41 years old and reaching 73% of change by 20 years old. Genu best fits a quadratic, peaking at 53 years old (53% change by 20 years). CST best fits a Poisson, with peak at 53 years old (61% change by 20 years). While cingulum best fits a Poisson, reaching a peak at 47 years old and reaching 67% of this ppb change by 20 years old (Figure 3 and Table 3). All best fits and respective parameters for $\chi_{\text{para}}$ and $\left|\chi_{\mathrm{dia}}\right|$ for WM regions can be found in Table 4.

**TABLE 3:** Values predicted by curves best fits for paramagnetic χ and absolute diamagnetic χ maps within white matter regions.

		Value at 5 years (ppb)	Age at peak value (years)	Value at 90 years (ppb)	Age at 90% of total change from 5 years to peak (years)^a^	% change from 5 to 20 years (%)
Paramagnetic χ	Genu					
	χ‐separation^b^					
	χ‐sepnet	23.0	29	24.7	21	85.9
	APART‐QSM^c^	13.5	19	15.8	14	
	Body of CC					
	χ‐separation	14.1	57	16.1	40	49.5
	χ‐sepnet	19.2	36	18.5	24	80.2
	APART‐QSM	8.9		10.0	81	17.6
	Splenium					
	χ‐separation	17.6	61	22.3	43	46.3
	χ‐sepnet^c^	20.3	15	23.0	12	
	APART‐QSM	9.6		12.8	82	17.6
	CST					
	χ‐separation	13.2	65	20.9	46	43.7
	χ‐sepnet	19.2		22.6	82	17.6
	APART‐QSM	8.5	69	11.8	49	41.2
	Cingulum					
	χ‐separation	10.4		21.0	40	62.8
	χ‐sepnet	19.9		23.4	18	93.3
	APART‐QSM	7.9	69	11.7	49	41.4
|Diamagnetic χ|	Genu of CC					
	χ‐separation	25.8	53	27.1	38	53.0
	χ‐sepnet	26.2	39	27.9	26	76.7
	APART‐QSM	13.2		16.6	58	44.6
	Body					
	χ‐separation	25.2	62	32.1	44	45.8
	χ‐sepnet	31.3	55	32.7	39	50.9
	APART‐QSM	15.4	80	19.9	56	36.0
	Splenium					
	χ‐separation	26.6	41	33.7	27	73.6
	χ‐sepnet	34.1	29	34.1	20	89.6
	APART‐QSM	16.6	38	18.8	26	77.0
	CST					
	χ‐separation	21.1	53	31.2	34	61.7
	χ‐sepnet	29.2	50	34.5	33	64.2
	APART‐QSM	15.0	49	21.4	32	65.5

Cingulum					

χ‐separation	15.1	47	21.3	31	67.1

χ‐sepnet^b^					

APART‐QSM	9.7		12.6	44	58.5

Abbreviations: CC, Corpus callosum; CST, Corticospinal tract.

^a^ For fits that do not have a peak, the 90% of total change was calculated between 5 to 90 years old.

^b^ Genu paramagnetic χ best fit with χ‐separation and cingulum diamagnetic χ best fit with χ‐sepnet were not significant.

^c^ For Genu paramagnetic χ best fit with APART‐QSM and splenium paramagnetic χ best fit with χ‐sepnet, the trajectory reached a peak at an age younger than 20 years old.

**TABLE 4:** Parameter values for curves best fits for paramagnetic χ and absolute diamagnetic χ maps within white matter regions.

		Fit	A	B	C	D	E
Paramagnetic χ	Genu						
	χ‐separation^a^						
	χ‐sepnet	Quadratic	23.15	−0.03	5.21E−04		
	APART‐QSM	Quadratic	13.53	−0.02	4.68E−04		
	Body of CC						
	χ‐separation	Quadratic	13.46	0.14	−1.24E−03		
	χ‐sepnet	Poisson	20.13			−0.22	−0.03
	APART‐QSM	Linear	8.87	0.01			
	Splenium						
	χ‐separation	Quadratic	16.44	0.25	−2.02E−03		
	χ‐sepnet	Quadratic	20.38	−0.02	4.94E−04		
	APART‐QSM	Linear	9.43	0.04			
	CST						
	χ‐separation	Quadratic	11.65	0.33	−2.55E−03		
	χ‐sepnet	Linear	18.95	0.04			
	APART‐QSM	Quadratic	7.88	0.12	−8.98E−04		
	Cingulum						
	χ‐separation	Exponential	21.06			−14.78	−0.07
	χ‐sepnet	Exponential	23.36			−8.60	−0.18
	APART‐QSM	Quadratic	7.17	0.14	−1.05E−03		
|Diamagnetic χ|	Genu						
	χ‐separation	Quadratic	25.02	0.16	−1.54E−03		
	χ‐sepnet	Poisson	24.61			0.37	−0.03
	APART‐QSM	Exponential	16.79			−4.33	−0.04
	Body of CC						
	χ‐separation	Quadratic	23.56	0.35	−2.82E−03		
	χ‐sepnet	Quadratic	30.71	0.12	−1.10E−03		
	APART‐QSM	Quadratic	14.81	0.13	−8.11E−04		
	Splenium						
	χ‐separation	Poisson	20.95			1.27	−0.02
	χ‐sepnet	Poisson	31.25			0.68	−0.03
	APART‐QSM	Poisson	14.28			0.53	−0.03
	CST						
	χ‐separation	Poisson	17.21			0.86	−0.02
	χ‐sepnet	Poisson	26.85			0.51	−0.02
	APART‐QSM	Poisson	11.95			0.67	−0.02
	Cingulum						

χ‐separation	Poisson	11.86			0.72	−0.02

χ‐sepnet^a^						

APART‐QSM	Exponential	12.60			−3.82	−0.06

*Note:* Fits were modeled based on the A–E parameters as follows: Linear = A+B·age; Quadratic = $A+B\cdot \mathrm{age}+C\cdot {\mathrm{age}}^{2}$; Exponential = $A+D\cdot e^{E\cdot \mathrm{age}}$; Poisson = $A+D\cdot \mathrm{age}\cdot e^{E\cdot \mathrm{age}}$.

Abbreviations: CC, Corpus callosum; CST, Corticospinal tract.

^a^ Genu paramagnetic χ best fit with χ‐separation and cingulum diamagnetic χ best fit with χ‐sepnet were not significant.

Focusing on the susceptibility values of the lifespan curves, WM ROIs had a smaller ppb range than DGM ROIs. The variation of susceptibility source content in WM ROIs was within a maximum range of ~15 ppb (for splenium $\left|\chi_{\mathrm{dia}}\right|$) across the lifespan, while DGM ROIs, on average, went through a change of 48 ppb in paramagnetic content and 15 ppb in diamagnetic content. Within DGM ROIs, caudate and putamen showed similar trends, thalamus showed the lowest paramagnetic values (mean of 23 ppb from 5 to 90 years) and higher absolute diamagnetic values (21 ppb), while globus pallidus had the highest values within $\chi_{\text{para}}$ (120 ppb). Regarding the WM ROIs, for most of the lifespan, splenium was the region with higher diamagnetic content (37 ppb), followed by CST and body of CC (about 32 ppb), genu (28 ppb) and cingulum (22 ppb). While for the paramagnetic content, splenium showed the highest concentration (22 ppb) and body of CC the lowest (16 ppb).

### 3.4 Comparisons Between Different Susceptibility Source Separation Methods

**FIGURE 4:**
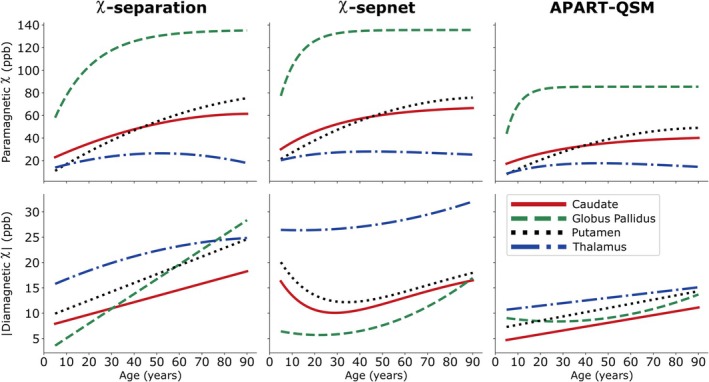
Best‐fit curves in deep gray matter regions of paramagnetic χ (top row) and absolute diamagnetic χ (bottom row) maps from χ‐separation, χ‐sepnet and APART‐QSM.

**FIGURE 5:**
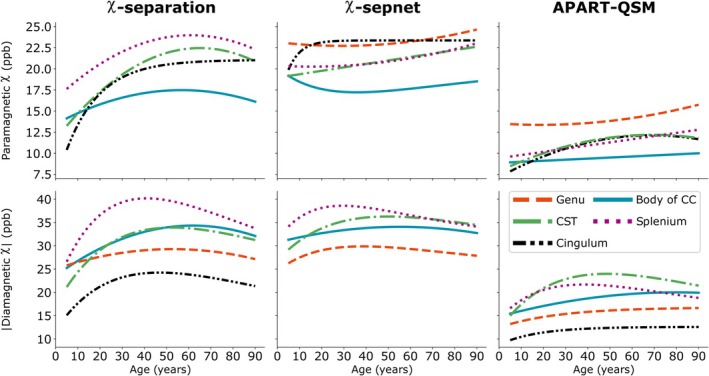
Best‐fit curves in white matter regions of paramagnetic χ (top row) and absolute diamagnetic χ (bottom row) maps from χ‐separation, χ‐sepnet and APART‐QSM. Genu paramagnetic χ best fit with χ‐separation and cingulum diamagnetic χ best fit with χ‐sepnet were not significant, so no curve shown. CC, Corpus callosum; CST, Corticospinal tract.

Regarding the results of the other SSS methods, with default initial QSM, all best fits were significant (*p* < 0.05), except for cingulum $\left|\chi_{\mathrm{dia}}\right|$ with χ‐sepnet. For χ‐separation_STAR and APART‐QSM_MEDI, most best fits were significant (p < 0.05), except for the $\chi_{\text{para}}$ genu with χ‐separation_STAR. Overall, the data points were visually more spread with χ‐separation across the whole lifespan; however, in later life, a spread in the data can be seen with all evaluated methods, especially in DGM ROIs (Figures 2 and 3 and Figures S3, 4, 5, and 6). Figures 4 and 5 show a summary of the best fits for all analyzed SSS methods with DGM and WM ROIs, respectively. Regarding the different methods, $\chi_{\text{para}}$ had similar trends for DGM ROIs. APART‐QSM showed consistently lower ppb values for both $\chi_{\text{para}}$ and $\left|\chi_{\mathrm{dia}}\right|$, regardless of ROI. χ‐sepnet showed different results for young subjects than the other two methods, more evident in $\left|\chi_{\mathrm{dia}}\right|$ for DGM ROIs or $\chi_{\text{para}}$ for WM ROIs. Regarding the susceptibility values (in ppb), the relationship between different ROIs was mostly not affected by the SSS methods, meaning that, even though susceptibility values and trends changed for each method, the relationship between different ROIs was kept regardless of SSS method. The exceptions were: globus pallidus with $\left|\chi_{\mathrm{dia}}\right|$, which had ppb values lower than the other DGM ROIs with χ‐sepnet, but comparable values to the other ROIs with the other two SSS methods; and WM ROIs with $\chi_{\text{para}}$, which showed different patterns for each SSS method, and the only similarity was the body of CC as the lowest region on ppb for all methods.

The evaluation of the linear correlation between the three SSS methods showed that χ‐separation and APART‐QSM are strongly linearly correlated with each other, with all R coefficients exceeding 0.8, with the exception of $\left|\chi_{\mathrm{dia}}\right|$ globus pallidus (R = 0.69) and $\chi_{\text{para}}$ genu (R = 0.74). Χ‐sepnet also showed a strong correlation (R > 0.6) to both methods in most of the comparisons, however, with lower R coefficients, as 12 out of the 18 evaluated maps had an R lower than 0.8 when comparing χ‐sepnet to χ‐separation, while to APART‐QSM, 10 maps were below this threshold. Overall, when comparing χ‐sepnet maps to the other methods, $\left|\chi_{\mathrm{dia}}\right|$ maps had lower R than $\chi_{\text{para}}$. $\left|\chi_{\mathrm{dia}}\right|$ putamen was the map with the lowest R when comparing χ‐sepnet to χ‐separation (0.4) and APART‐QSM (0.3), followed by $\left|\chi_{\mathrm{dia}}\right|$ caudate (0.55, comparing to APART‐QSM) and $\chi_{\text{para}}$ body of CC (0.59, comparing to χ‐separation). All calculated R coefficients are shown in Table S1.

### 3.5 Influence of Relaxometric Constant

The fitting of the two ROIs revealed that the change in the primary component ($\chi_{\text{para}}$ for caudate and $\chi_{\mathrm{dia}}$ for splenium) generally follows the equation χ = 0.5 * R2′ / D_r_ ± 0.5 * QSM, where the fitted parameters *a* and *b* were related to QSM and R2′ values, respectively. For example, considering χ‐separation, using the original D_r_ value (137 Hz/ppm), the mean $\chi_{\text{para}}$ within Caudate was 30.5 ppb, while with a D_r_ close to double the original one (275 Hz/ppm), this mean value dropped to 12.8 ppb. For the secondary component, the change was still closely related to R2′ but with a smaller contribution from QSM.

All $\chi_{\text{para}}$ and $\left|\chi_{\mathrm{dia}}\right|$ lifespan best fits were significant (*p* < 0.05) with all evaluated D_r_ values (50, 114, 137, 230 and 323.5 Hz/ppm) for Caudate and Splenium ROIs. The lifespan trajectories (Figure S7) showed that the shape of the curves was generally retained with different D_r_ values used as input; however, both $\chi_{\text{para}}$ and $\left|\chi_{\mathrm{dia}}\right|$ maps consistently increased with smaller D_r_ values. Regarding the different SSS methods, χ‐separation and APART‐QSM showed similar results for variable D_r_ choices, while χ‐sepnet had a much smaller range of change.

### 3.6 Influence of Initial QSM

QSM calculated with MEDI showed higher absolute ppb values than with STAR (Figure S8). This translated into the SSS outputs, but differently for each method. χ‐separation SSS outputs showed similar results regardless of the QSM input, while APART‐QSM had a stronger dependence on the initial QSM. More specifically, for DGM ROIs, χ‐separation and χ‐separation_STAR showed similar trajectories across the lifespan, except for the $\chi_{\text{para}}$ globus pallidus, while with APART‐QSM, most ROIs showed slightly different curve shapes and amplitudes depending on the used dipole inversion method, especially on $\left|\chi_{\mathrm{dia}}\right|$, and more clearly seen with globus pallidus and putamen (Figure S9). A similar pattern was seen in the WM ROIs. More specifically, genu and CST showed the greatest difference when comparing APART‐QSM_MEDI to APART‐QSM (Figure S10).

### 3.7 Comparison Between Susceptibility Source Separation and QSM Only

All QSM best fits were significant (*p* < 0.05) with MEDI and STAR, except for cingulum with MEDI (Figure S8). Overall, the dominant susceptibility source of each ROI (paramagnetic for DGM and diamagnetic for WM) had similar trajectory to QSM‐only maps; however, the absolute susceptibility value was lower in QSM, and the information from the secondary source was not seen in QSM. For example, in caudate and putamen, the separated maps unveiled the changes in diamagnetic content with age. For the thalamus, both $\chi_{\text{para}}$ and $\left|\chi_{\mathrm{dia}}\right|$ values were at least 10 ppb for most of the lifespan, in contrast to QSM, which had values close to 0 ppb (Figure 4 and Figure S8). For WM ROIs, QSM values are negative, indicative of dominant diamagnetic content, with the use of SSS also demonstrating the paramagnetic sources (Figure 5 and Figure S8). Predicted values for the QSM best fits with MEDI and STAR can be found in Table S2, while all best fits and respective parameters can be found in Table S3.

### 3.8 Relaxation Maps Lifespan Trajectories

All best fits were significant (*p* < 0.05) for all relaxation maps (R2′ and R2*). Predicted values for the relaxation maps best fits can be found in Table S4, while all best fits and respective parameters can be found in Table S5. For DGM R2*, the lifespan curves showed almost the same trends as seen in a previous study [33], which reported lifespan curves within DGM regions for a similar dataset (combining the 339 subjects included in this work, with an additional 159 subjects); small differences between the two studies are due to the fact that, in the current study, best fits were chosen for each trajectory individually instead of using cubic fits only. In DGM R2′, most of the evaluated ROIs showed an increasing trend throughout the lifespan, except for Thalamus, which initially increased with age, reached a peak at 61 years old, and decreased in later ages (Figure S11); increasing trends with age for these ROIs were also seen in a previous study [15] that reported DGM R2′ lifespan curves, in addition to R2*. In WM, all of the ROIs showed an increasing trend in early ages, reached a peak around 50 and 40 years old, for R2′ and R2*, respectively, and then decreased with age, except for Genu R2′, which showed a linear increase with age. DGM ROIs showed higher values than WM in R2* and in R2′, which is expected as these regions are rich in iron, which is a strong susceptibility source; DGM values ranged, for most of the lifespan, from 5 to 20 Hz for R2′ (which were slightly lower than what was found in a previous study [15], possibly due to R2 underestimation causing R2′ overestimation in their work) and from 15 to 40 Hz for R2* (which were in agreement with previous reports [15, 33]), while for WM these values were from 4 to 9 Hz and 17 to 23 Hz, respectively.

## 4 Discussion

This work analyzed healthy lifespan trajectories from paramagnetic and diamagnetic sources in the human brain by plotting and fitting the data from three SSS methods (χ‐separation [23], χ‐sepnet [24] and APART‐QSM [25]) within white and gray matter regions against age. The results showed how paramagnetic and diamagnetic susceptibility sources vary with age in the healthy brain, how different SSS methods affect lifespan results, and the influence that early age data has on these trajectories.

Most of the SSS paramagnetic and diamagnetic lifespan trajectories were in agreement with known iron and myelin changes with age, respectively. Throughout life, iron concentration is expected to increase with age in most regions of the healthy human brain [4], while myelin content increases with age until it peaks, around 40 to 50 years old, followed by a decrease with age in WM and by a smaller decrease in DGM [2]. Χ‐separation was successful in showing paramagnetic trajectories that can be related to known iron changes with age in all analyzed regions. Globus pallidus paramagnetic best fits were exponential and thalamus showed a quadratic pattern, as seen in previous literature [4], while caudate and thalamus trajectories showed the expected increase in paramagnetic content throughout the lifespan, even though their best fits were not the same as found in a previous histology study [4]. In addition, in white matter, diamagnetic curves were in agreement with known myelination and myelin loss periods across the lifespan; more specifically, genu, splenium, and cingulum diamagnetic maps with SSS showed trajectories similar to past MWF studies [2, 46].

As for the DGM diamagnetic trajectories, χ‐separation did not show the expected decrease in diamagnetic content due to myelin loss later in life; instead, it steadily increased with aging. Although this late life increase is on the order of 5 ppb, which is less than the reliability coefficient [39] of the methods, this trend was also found in all previous SSS lifespan studies [29, 30, 31]. Explanation for this increase may relate to the fact that DGM is highly rich in iron and generally increases with age, which may be overwhelming the much smaller diamagnetic sources in the modeling process. As SSS relies on the assumption that the sum of absolute $\chi_{\text{para}}$ and $\chi_{\mathrm{dia}}$ is related to R2′ by a constant (D_r_), in areas where R2′ may be overwhelming or inaccurately high, the model would force both $\chi_{\text{para}}$ and $\left|\chi_{\mathrm{dia}}\right|$ to be overestimated, as the difference between them is fixed by the net susceptibility value. Additionally, the same D_r_ value is used for para and diamagnetic susceptibilities, which prevents the model from adjusting the values for each source independently. Hence, this may present a limitation of the method and further studies are warranted, especially on the estimation of independent D_r_s for each susceptibility source. Alternatively, calcification, which may occur in the DGM at later ages, may be increasing diamagnetic content with aging, resulting in a ppb increase in $\left|\chi_{\mathrm{dia}}\right|$. More research is needed to determine whether this increase in the diamagnetic component in DGM in later life is only a limitation of current SSS methodology or also has partial biological underpinnings.

The inclusion of children in this work contributed to different lifespan trajectories in comparison to three previous SSS studies [29, 30, 31] that included only adults or very few children (Lao et al.: small cohort 3 from ages 4–39 years). In some ROIs, the three previous studies reported linear trajectories or no significant changes with age; however, with the inclusion of young subjects, different patterns were seen, as increases in paramagnetic and diamagnetic content occur in early ages. For example, in the CST, Lao et al. found an almost constant linear trajectory for diamagnetic susceptibility sources with both cohorts; in contrast, our results showed a steep increase in early life, a peak at 30 years old, followed by a smooth decrease that starts at 50 years old. These findings highlight the large changes in susceptibility sources from childhood to mid‐life.

Separated $\chi_{\text{para}}$ and $\left|\chi_{\mathrm{dia}}\right|$ showed some advantages in showing the underlying biological processes throughout life when compared to QSM‐only. Within WM ROIs, myelin concentrations are expected to follow a quadratic trajectory, increasing with development, peaking during midlife and then decreasing with aging, while iron concentrations are expected to increase with age. However, with QSM‐only, the trajectories highlight the dominant diamagnetic changes only, which can be associated with changes in myelin, while with SSS, both iron and myelin processes can be identified independently. Regarding DGM ROIs, SSS methods were more valuable than QSM‐only in the thalamus, where both paramagnetic and diamagnetic sources are prominent throughout the lifespan. However, within the other iron‐rich DGM ROIs (caudate, putamen and globus pallidus), $\chi_{\text{para}}$ was similar to QSM‐only and showed biologically expected trajectories dominated by iron. However, $\left|\chi_{\mathrm{dia}}\right|$ in these DGM regions showed unexpected trajectories, as diamagnetic content increased with age instead of levelling off after midlife due to myelin loss.

The three SSS methods produced different maps and different lifespan trajectories. For all ages, maps and ROIs, susceptibility maps acquired with APART‐QSM were lower than other SSS methods. This is due to the fact that APART‐QSM uses a higher D_r_ value (initially set to 323.5 Hz/ppm, and iteratively calculated to a final average D_r_ of 210 Hz/ppm and 269 Hz/ppm, for DGM and WM, respectively) than the fixed D_r_ in χ‐separation (137 Hz/ppm) and in χ‐sepnet (114 Hz/ppm), and as SSS models are solving for $R2^{\prime }=D_{r}\cdot \left(\left|\chi_{\text{para}}\right|+\left|\chi_{\mathrm{dia}}\right|\right)$, a higher D_r_ will result in lower values for the susceptibility sources maps, as demonstrated in this work, when different initial D_r_s were used as inputs for the 3 SSS methods. Χ‐sepnet showed an unexpected pattern for early ages; in most of the DGM ROIs, diamagnetic content showed a decrease, instead of the expected increasing values due to myelination in this period. This may be due to the fact that χ‐sepnet is a deep learning approach, which was trained in an older cohort (mean age of 25.5 years old), and might not perform the same way in a cohort aged far from the trained age range.

Χ‐separation showed more data spread in comparison to the other methods, which could be due to the use of CSF referencing in the χ‐separation model, which has been previously reported to increase data variability when compared to implicit referencing to the brain average [5]. In comparison to χ‐separation, the other evaluated methods had a smaller range of values in their outputs. This smaller range seen across the lifespan with the deep‐learning approach, χ‐sepnet, might be caused by a constraint in its results to the range of values with which it was trained, while APART‐QSM shows a smaller range due to the use of higher D_r_, previously discussed. Correlation testing showed that all methods had strong agreement with each other, although χ‐sepnet was less correlated to the other methods in diamagnetic measures.

The choice of dipole inversion method used to acquire the initial QSM guess influenced the SSS outputs. Χ‐separation was less sensitive to the initial QSM guess than APART‐QSM, because χ‐separation uses QSM only to initialize $\chi_{\text{para}}$ and $\chi_{\mathrm{dia}}$, while in APART‐QSM the cost function of the inverse model includes a term that enforces the sum of the separated maps to be as close as possible to the initial QSM. Similarly, the maps of the dominant sources for each ROI group ($\chi_{\text{para}}$ for DGM and $\left|\chi_{\mathrm{dia}}\right|$ for WM) showed equivalent results with APART‐QSM and APART‐QSM_MEDI. However, the trajectories with the secondary susceptibility source map ($\left|\chi_{\mathrm{dia}}\right|$ for DGM and $\chi_{\text{para}}$ for WM) were very different, most notably for $\left|\chi_{\mathrm{dia}}\right|$ in globus pallidus, where the choice of QSM method in the APART‐QSM method influenced the best curve shape. Even though the data points for $\left|\chi_{\mathrm{dia}}\right|$ in globus pallidus with APART‐QSM and APART‐QSM_MEDI showed a similar trend for portions of the lifespan, the APART‐QSM method had increased relative variability in late life versus younger ages (data not shown), leading to a quadratic best fit, while APART‐QSM_MEDI was exponential for this diamagnetic component. Note, however, this best curve for APART‐QSM varied by only ~5 ppb between age 5 and age 90.

Relaxation maps trajectories across the lifespan, especially R2′ maps, were useful for better understanding the SSS outputs. While QSM depends on the difference of $\chi_{\text{para}}$ and $\left|\chi_{\mathrm{dia}}\right|$, R2′ is dependent on the sum, which is the basis of all SSS methods. In general, the R2′ curves are reflective of the dominant susceptibility source, with SSS necessary to pull out the trends of the weaker susceptibility component.

Limitations of this work include the lack of the early‐development period from 0 to 5 years old, when substantial susceptibility changes occur [4, 47]. Another limitation is that the increasing diamagnetic susceptibility of DGM in late life is not fully understood; while we hypothesize method limitations or calcification as causes for this finding, further investigation is warranted. Additionally, the present study analyzed a cross‐sectional dataset, which shows trends that may be biased by a cohort effect without individual trajectories that may be particularly important for comparisons within patient studies [48, 49]. Lastly, the mismatch in spatial resolution between acquisitions may add variability due to the multiple interpolation and registration steps used in compiling the results, although this is unlikely to affect final lifespan curves given the large number of subjects averaged.

## 5 Conclusion

Susceptibility source separation demonstrated distinct paramagnetic and diamagnetic susceptibility trajectories across the lifespan. By using ages 5–90 years, the trajectories capture early brain maturation and late life declines, with early ages being important for defining the fitted trajectory. Independent paramagnetic and diamagnetic trajectories were most valuable over standard QSM in regions without a highly dominant susceptibility component, where both iron and myelin trajectories can be distinguished. Results using three common SSS methods showed different values and shapes in lifespan trajectories depending on the method chosen.

## Author Contributions

**T.B.O.A.:** methodology, formal analysis, investigation, writing – original draft, visualization. **N.N.:** investigation, software, writing – review and editing. **P.S.:** data acquisition, data curation, software, writing – review and editing. **C.B.:** conceptualization, project administration, writing – review and editing, funding acquisition. **A.H.W.:** conceptualization, supervision, project administration, writing – review and editing, funding acquisition.

## Funding

This work was supported by the Canadian Institutes of Health Research (A.H.W.) and University Hospital Foundation (C.B.).

## Conflicts of Interest

The authors declare no conflicts of interest.

## Supporting information


**Figure S1:** Relaxation maps (R2′ and R2*) and QSM (MEDI and STAR) from 5 representative subjects across the lifespan.
**Figure S2:** Data points and best‐fit curves for χ‐separation with males and females fitted separately for the cases that showed a statistically significant difference between sexes. Left and right hemispheres were averaged for each region.
**Figure S3:** Data points and best‐fit curves within deep gray matter regions for χ‐sepnet χpara and χdia maps. Left and right hemispheres were averaged for each region. Males (blue) and females (red) were combined for the curve fitting.
**Figure S4:** Data points and best‐fit curves within white matter regions for χ‐sepnet χpara and χdia maps. Left and right hemispheres were averaged for each region. Males (blue) and females (red) were combined for the curve fitting. Cingulum absolute diamagnetic χ best fit was not significant, so no curve shown. CC, Corpus callosum.
**Figure S5:** Data points and best‐fit curves within deep gray matter regions for APART‐QSM χpara and χdia maps. Left and right hemispheres were averaged for each region. Males (blue) and females (red) were combined for the curve fitting.
**Figure S6:** Data points and best‐fit curves within white matter regions for APART‐QSM χpara and χdia maps. Left and right hemispheres were averaged for each region. Males (blue) and females (red) were combined for the curve fitting. CC, Corpus callosum.
**Figure S7:** Effect of varying relaxometric constant (Dr) on best‐fit curves in Caudate (top panel) and Splenium (bottom panel) for paramagnetic χ and absolute diamagnetic χ maps, from χ‐separation, χ‐sepnet and APART‐QSM. Light green colored curves correspond to the curves with the original Dr used for that method (137 Hz/ppm for χ‐separation, 114 Hz/ppm for χ‐sepnet and 323.5 Hz/ppm for the APART‐QSM initial guess). Shaded areas show 95% confidence interval, color‐coded to each curve.
**Figure S8:** Best‐fit curves for QSM in deep gray matter (left column) and white matter (right column) regions, with MEDI (top row) and STAR (bottom row). Shaded areas show 95% confidence interval, color‐coded to each curve. Cingulum QSM MEDI best fit was not significant, so no curve shown. CC, Corpus callosum; CST, Corticospinal tract.
**Figure S9:** Effect of QSM method on deep gray matter best fit curves of paramagnetic χ and absolute diamagnetic χ maps, from χ‐separation and APART‐QSM with MEDI or STAR QSM as first guess inputs. Shaded areas show 95% confidence interval, color‐coded to each curve. The paramagnetic χ and absolute diamagnetic χ curves from χ‐separation with MEDI and from APART‐QSM with STAR are also shown in Figure 4.
**Figure S10:** Effect of QSM method on white matter best‐fit curves of paramagnetic χ and absolute diamagnetic χ maps, from χ‐separation and APART‐QSM with MEDI and STAR QSM as first guess inputs. Shaded areas show 95% confidence interval, color‐coded to each curve. Genu paramagnetic χ best fits with χ‐separation (independent of first guess QSM) were not significant, so no curve shown. The paramagnetic χ and absolute diamagnetic χ curves from χ‐separation with MEDI and from APART‐QSM with STAR are also shown in Figure 5. CC, Corpus callosum; CST, Corticospinal tract.
**Figure S11:** Best‐fit curves in deep gray matter (left column) and white matter (right column) regions for relaxation maps: R2′ (top row) and R2* (bottom row). Shaded areas show 95% confidence interval, color‐coded to each curve. CC, Corpus callosum; CST, Corticospinal tract.
**Table S1:** Linear correlation between susceptibility source separation methods. Values shown are Pearson correlation coefficients for paramagnetic and diamagnetic χ for all ROIs and subjects. Values below 0.60 are in bold.
**Table S2:** QSM peak values and changes from 5 to 90 years from best‐fit curves.
**Table S3:** Parameter values for best‐fit curves for QSM.
**Table S4:** R2′ and R2* peak values and changes from 5 to 90 years from best‐fit curves.
**Table S5:** Parameter values for best‐fit curves for relaxation maps (R2′ and R2*).

## Data Availability

The measurements (mean value per subject with age and sex, for all reported regions and metrics—χ_para_, χ_dia_, QSM, R2′ and R2*) and analysis scripts of this study are available from the corresponding author upon request. Participant MRI images and maps are not publicly available due to ethical considerations.
