# Supplementary material for: Susceptibility Source Separation Unveils Paramagnetic and Diamagnetic Trajectories in Healthy Brains From 5 to 90 Years
Source: NMR Biomed. 2026 Jul 6;39(8):e70349. doi: 10.1002/nbm.70349 (PMC13334517; doi:10.1002/nbm.70349)
Supplement: Supplementary file 1 — Figure S1: Relaxation maps (R2′ and R2*) and QSM (MEDI and STAR) from 5 representative subjects across the lifespan. Figure S2: Data points and best‐fit curves for χ‐separation with males and females fitted separately for the cases that showed a statistically significant difference between sexes. Left and right hemispheres were averaged for each region. Figure S3: Data points and best‐fit curves within deep gray matter regions for χ‐sepnet χpara and χdia maps. Left and right hemispheres were averaged for each region. Males (blue) and females (red) were combined for the curve fitting. Figure S4: Data points and best‐fit curves within white matter regions for χ‐sepnet χpara and χdia maps. Left and right hemispheres were averaged for each region. Males (blue) and females (red) were combined for the curve fitting. Cingulum absolute diamagnetic χ best fit was not significant, so no curve shown. CC, Corpus callosum. Figure S5: Data points and best‐fit curves within deep gray matter regions for APART‐QSM χpara and χdia maps. Left and right hemispheres were averaged for each region. Males (blue) and females (red) were combined for the curve fitting. Figure S6: Data points and best‐fit curves within white matter regions for APART‐QSM χpara and χdia maps. Left and right hemispheres were averaged for each region. Males (blue) and females (red) were combined for the curve fitting. CC, Corpus callosum. Figure S7: Effect of varying relaxometric constant (Dr) on best‐fit curves in Caudate (top panel) and Splenium (bottom panel) for paramagnetic χ and absolute diamagnetic χ maps, from χ‐separation, χ‐sepnet and APART‐QSM. Light green colored curves correspond to the curves with the original Dr used for that method (137 Hz/ppm for χ‐separation, 114 Hz/ppm for χ‐sepnet and 323.5 Hz/ppm for the APART‐QSM initial guess). Shaded areas show 95% confidence interval, color‐coded to each curve. Figure S8: Best‐fit curves for QSM in deep gray matter (left column) and white matter (right colu [file NBM-39-e70349-s001.docx]

**SUPPLEMENTARY INFORMATION**

**FIGURES**


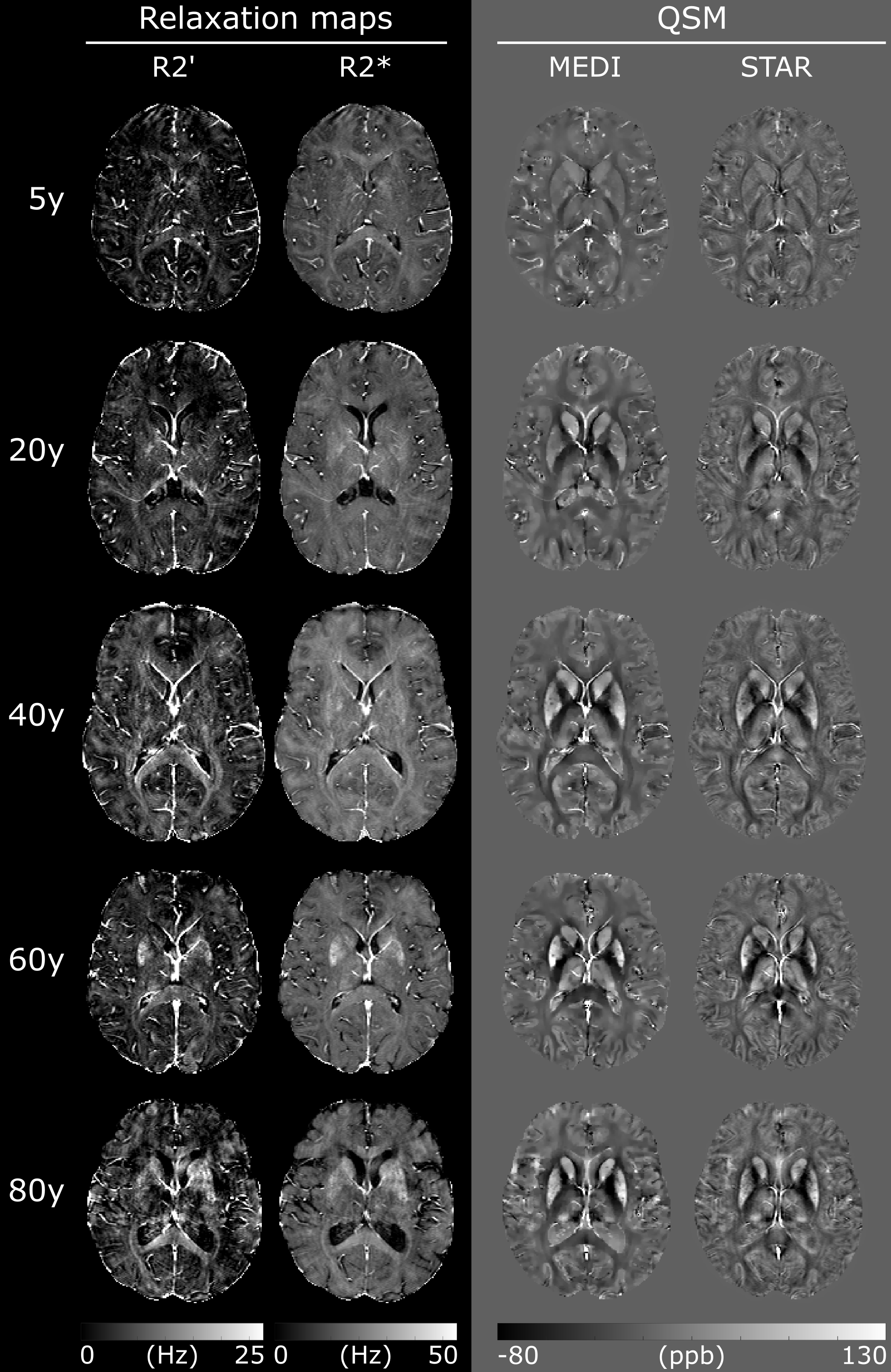


**Supplementary Figure 1.** Relaxation maps (R2’ and R2*) and QSM (MEDI and STAR) from 5 representative subjects across the lifespan.


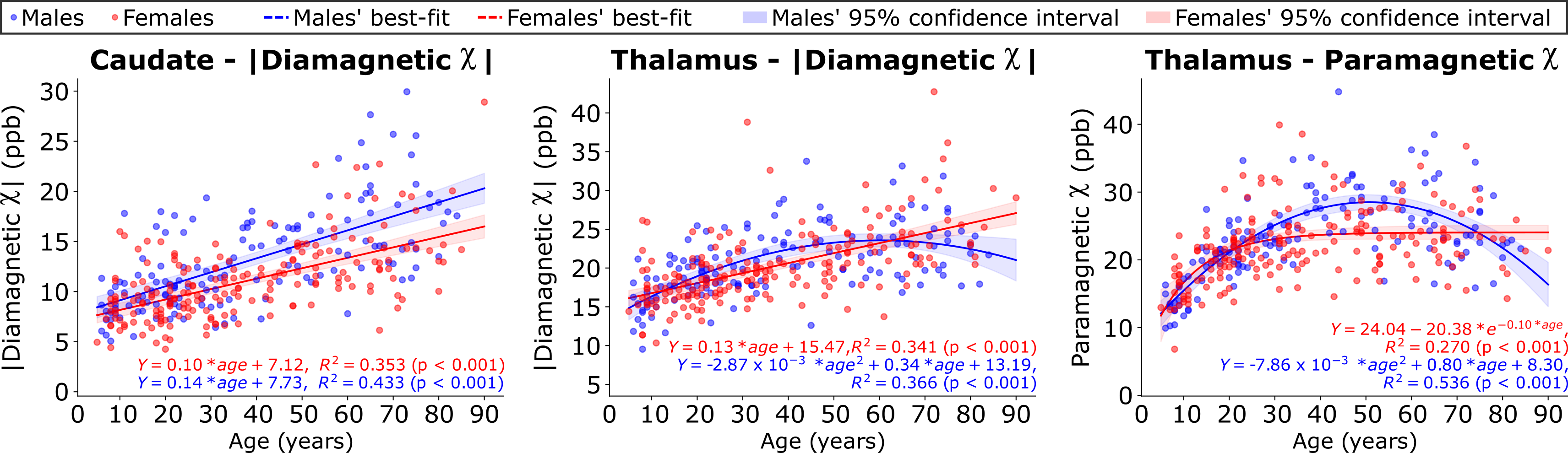


**Supplementary Figure 2.** Data points and best-fit curves for χ-separation with males and females fitted separately for the cases that showed a statistically significant difference between sexes. Left and right hemispheres were averaged for each region.


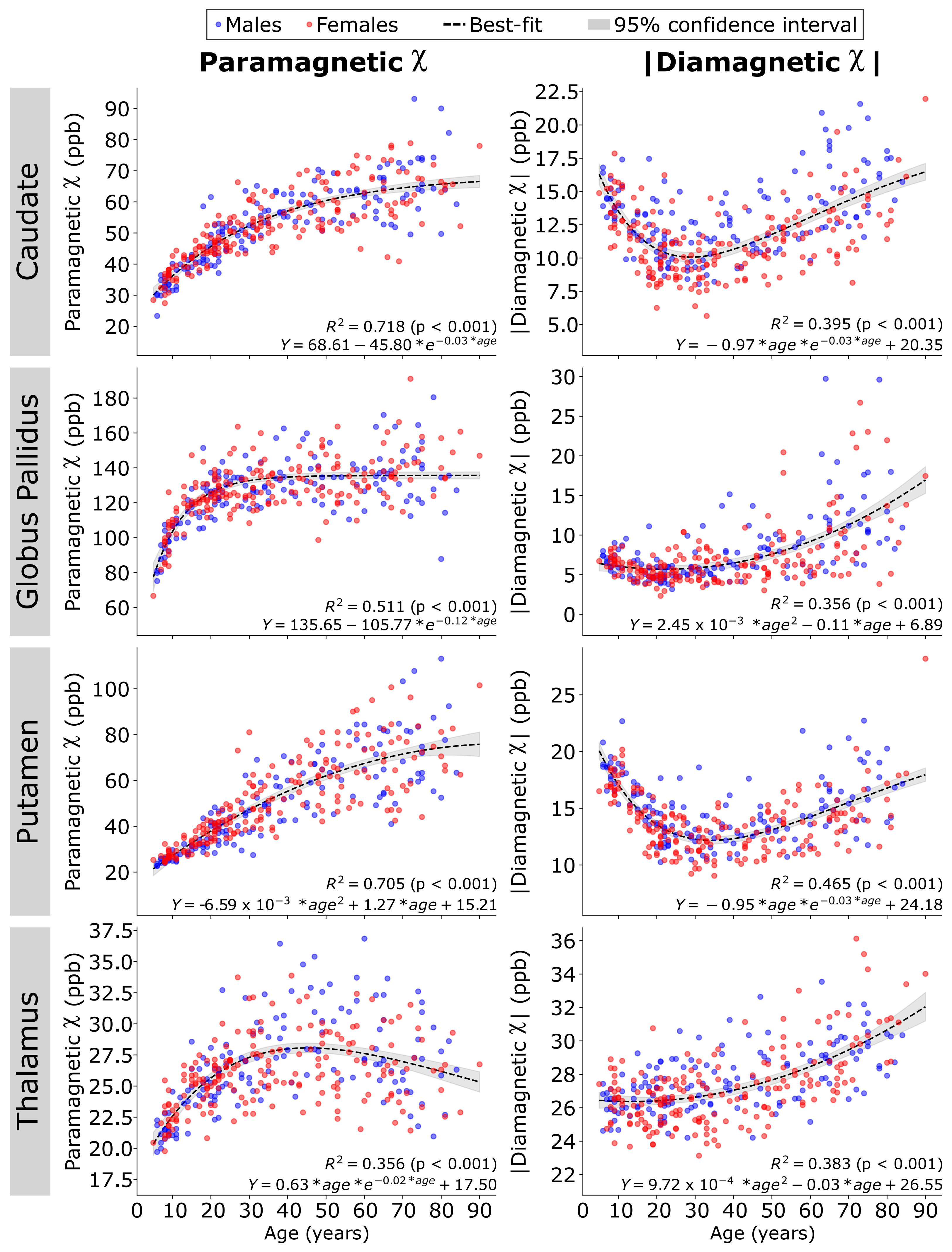


**Supplementary Figure 3.** Data points and best-fit curves within deep grey matter regions for χ-sepnet $\chi_{para}$ and $\left| \chi_{dia} \right|$ maps. Left and right hemispheres were averaged for each region. Males (blue) and females (red) were combined for the curve fitting.


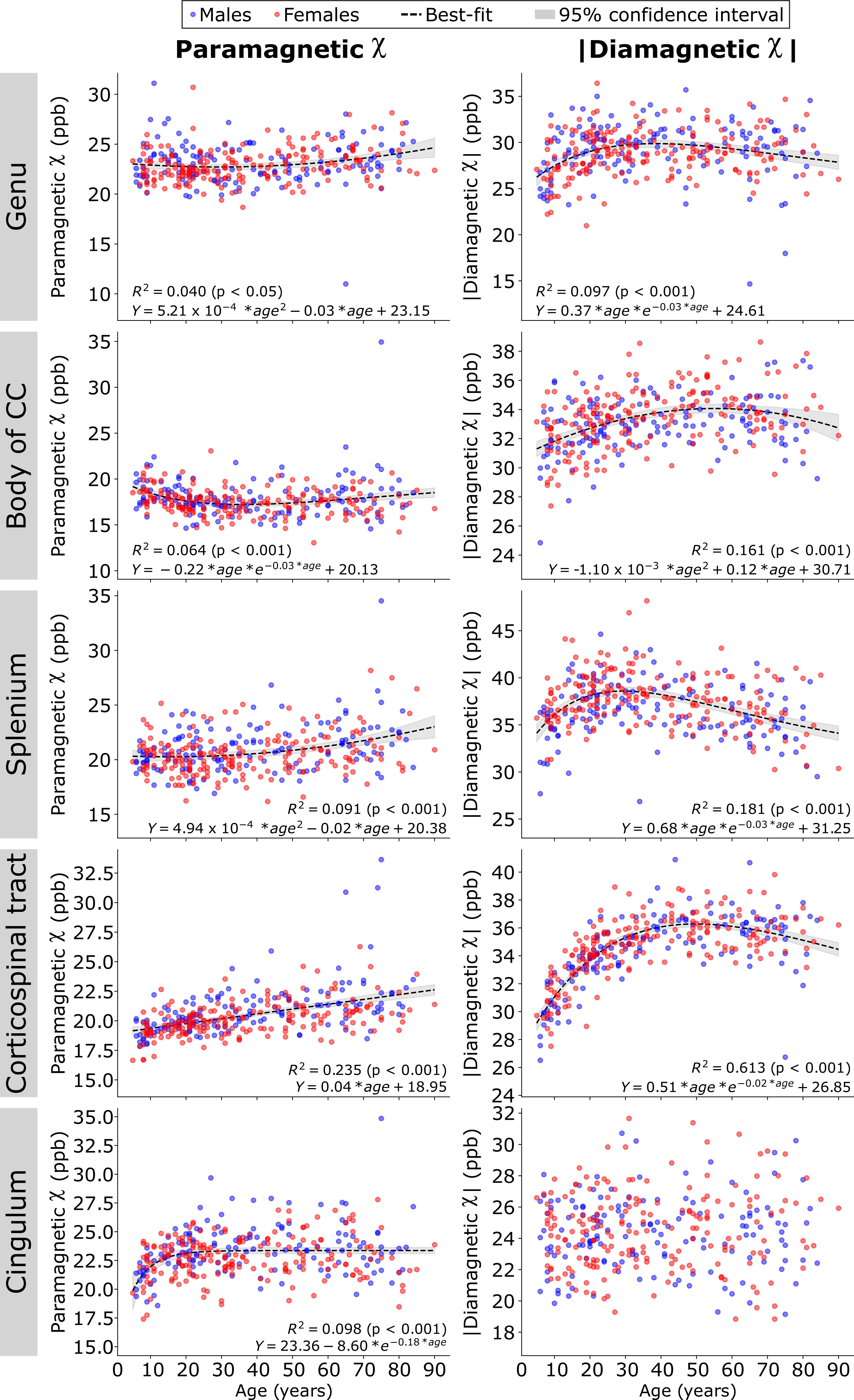


**Supplementary Figure 4.** Data points and best-fit curves within white matter regions for χ-sepnet $\chi_{para}$ and $\left| \chi_{dia} \right|$ maps. Left and right hemispheres were averaged for each region. Males (blue) and females (red) were combined for the curve fitting. Cingulum absolute diamagnetic χ best-fit was not significant, so no curve shown. CC, Corpus callosum.


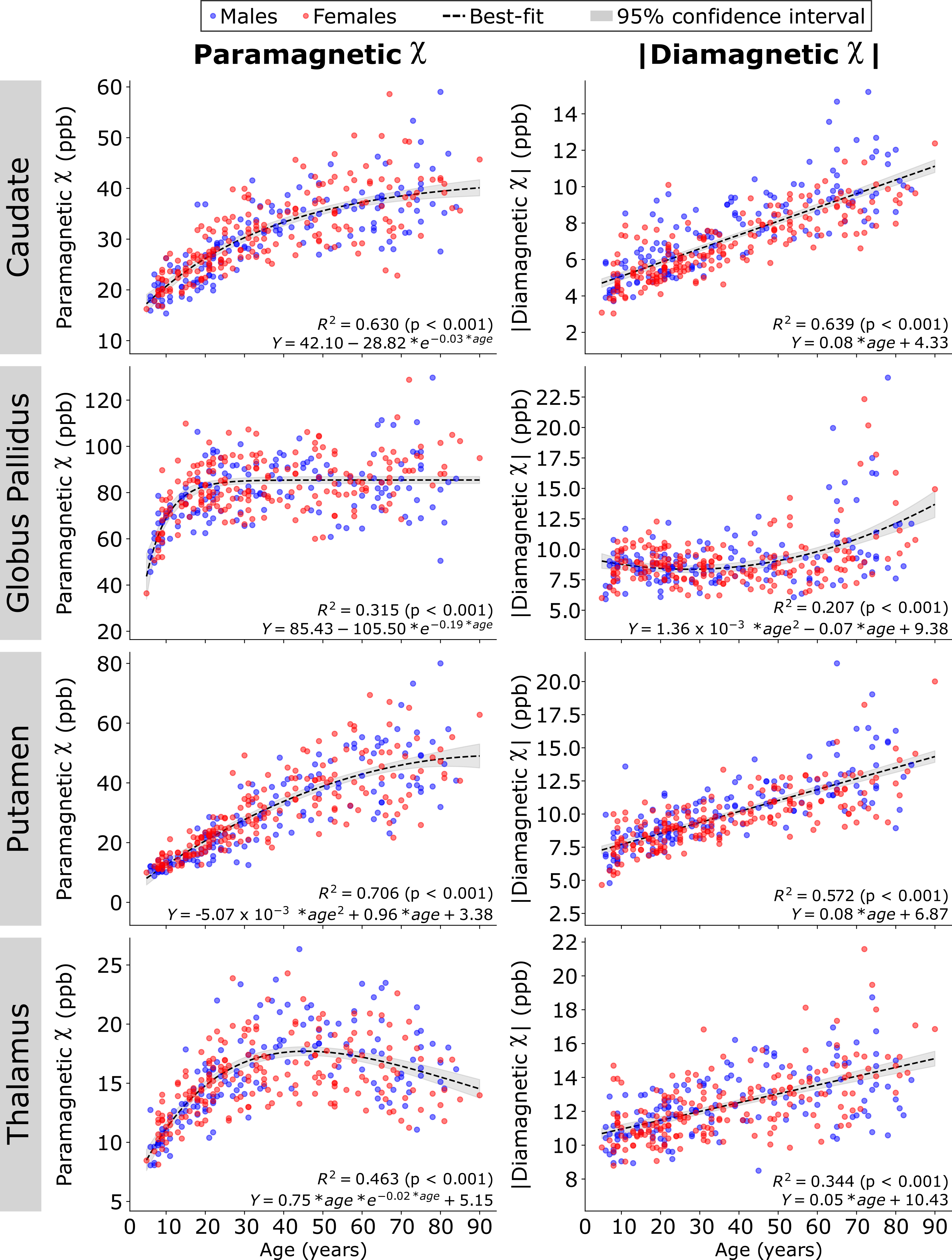


**Supplementary Figure 5.** Data points and best-fit curves within deep grey matter regions for APART-QSM $\chi_{para}$ and $\left| \chi_{dia} \right|$ maps. Left and right hemispheres were averaged for each region. Males (blue) and females (red) were combined for the curve fitting.


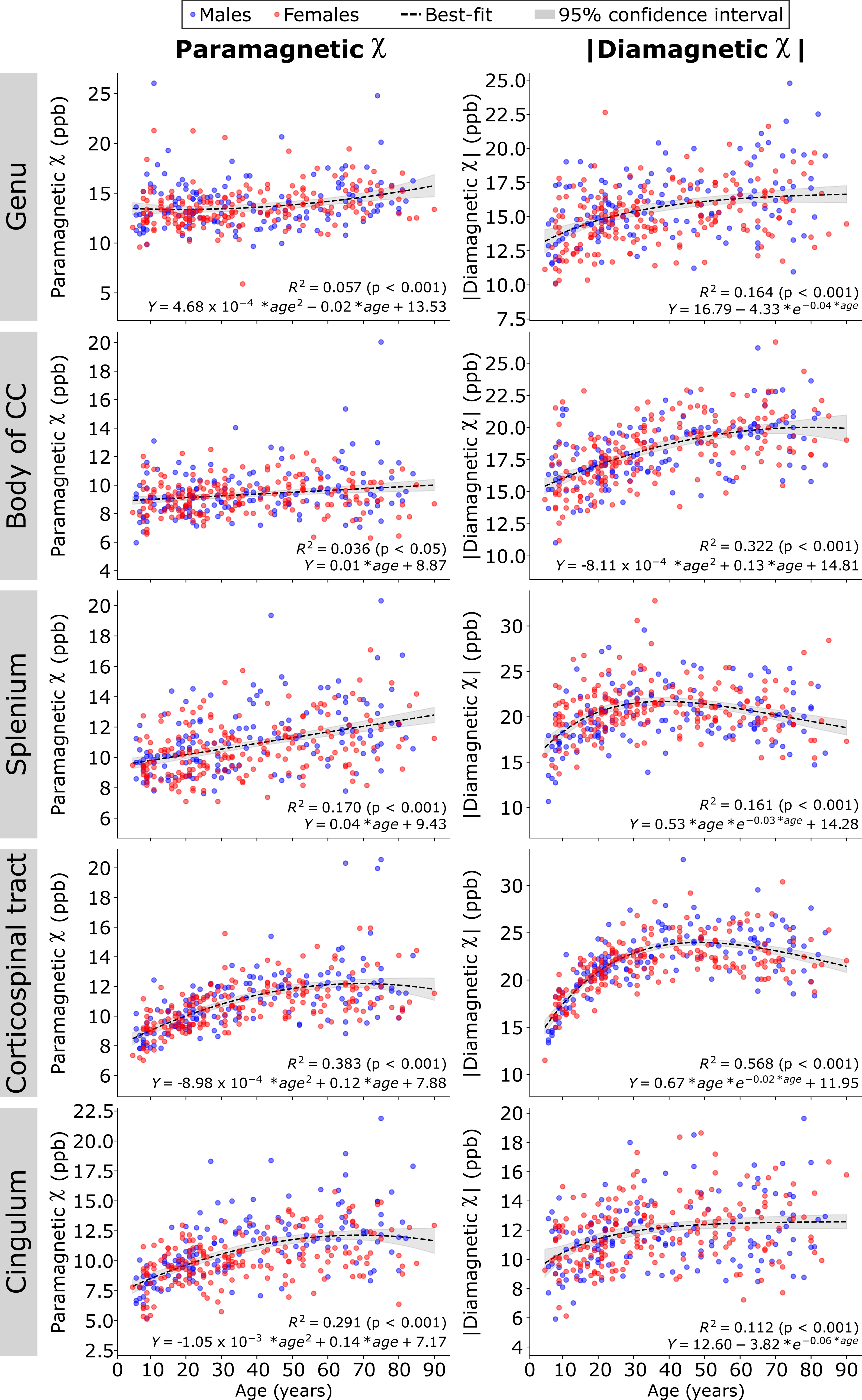


**Supplementary Figure 6.** Data points and best-fit curves within white matter regions for APART-QSM $\chi_{para}$ and $\left| \chi_{dia} \right|$ maps. Left and right hemispheres were averaged for each region. Males (blue) and females (red) were combined for the curve fitting. CC, Corpus callosum.


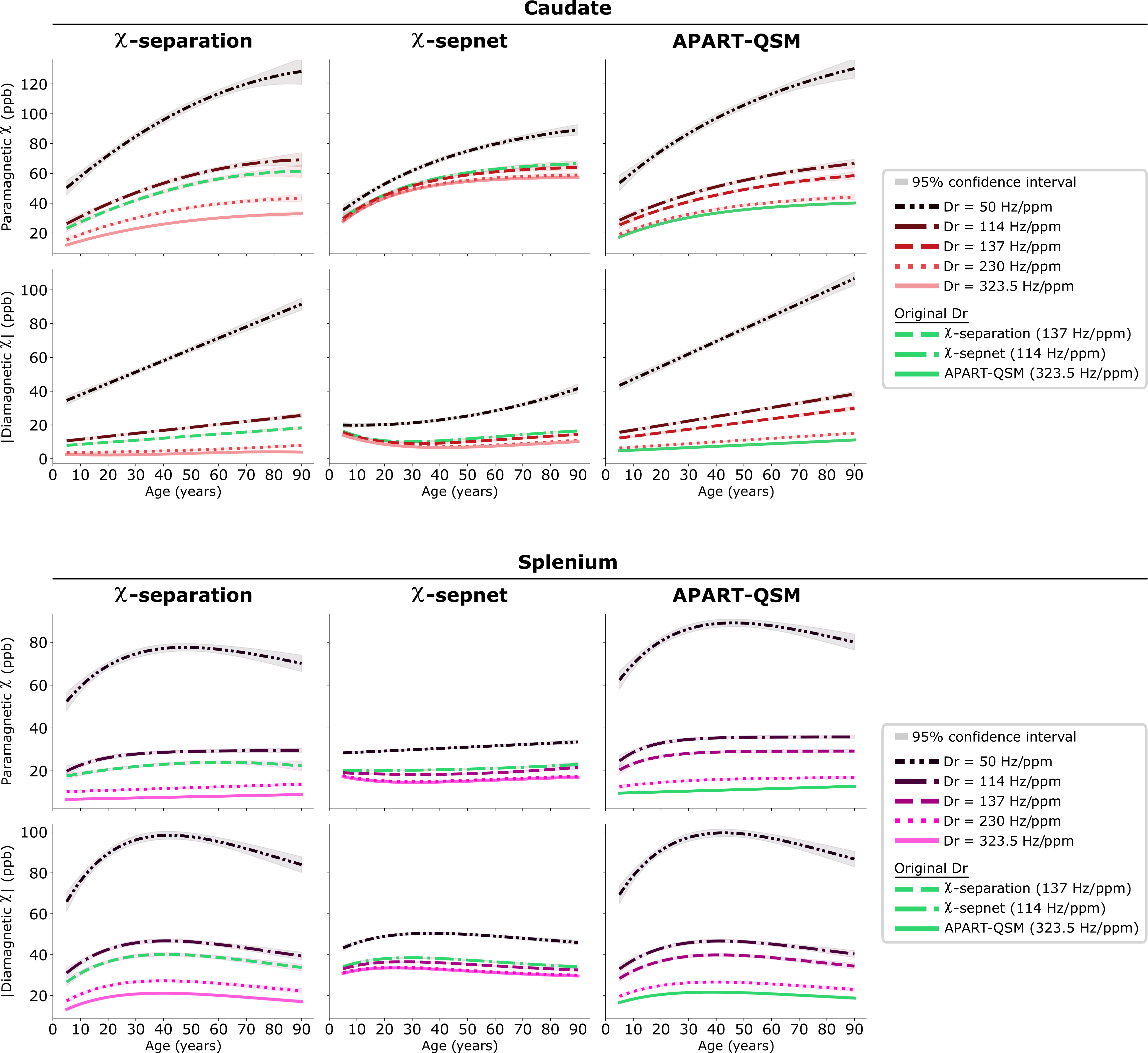


**Supplementary Figure 7.** Effect of varying relaxometric constant (D_r_) on best-fit curves in Caudate (top panel) and Splenium (bottom panel) for paramagnetic χ and absolute diamagnetic χ maps, from χ-separation, χ-sepnet and APART-QSM. Light green colored curves correspond to the curves with the original D_r_ used for that method (137 Hz/ppm for χ-separation, 114 Hz/ppm for χ-sepnet and 323.5 Hz/ppm for the APART-QSM initial guess). Shaded areas show 95% confidence interval, color-coded to each curve.


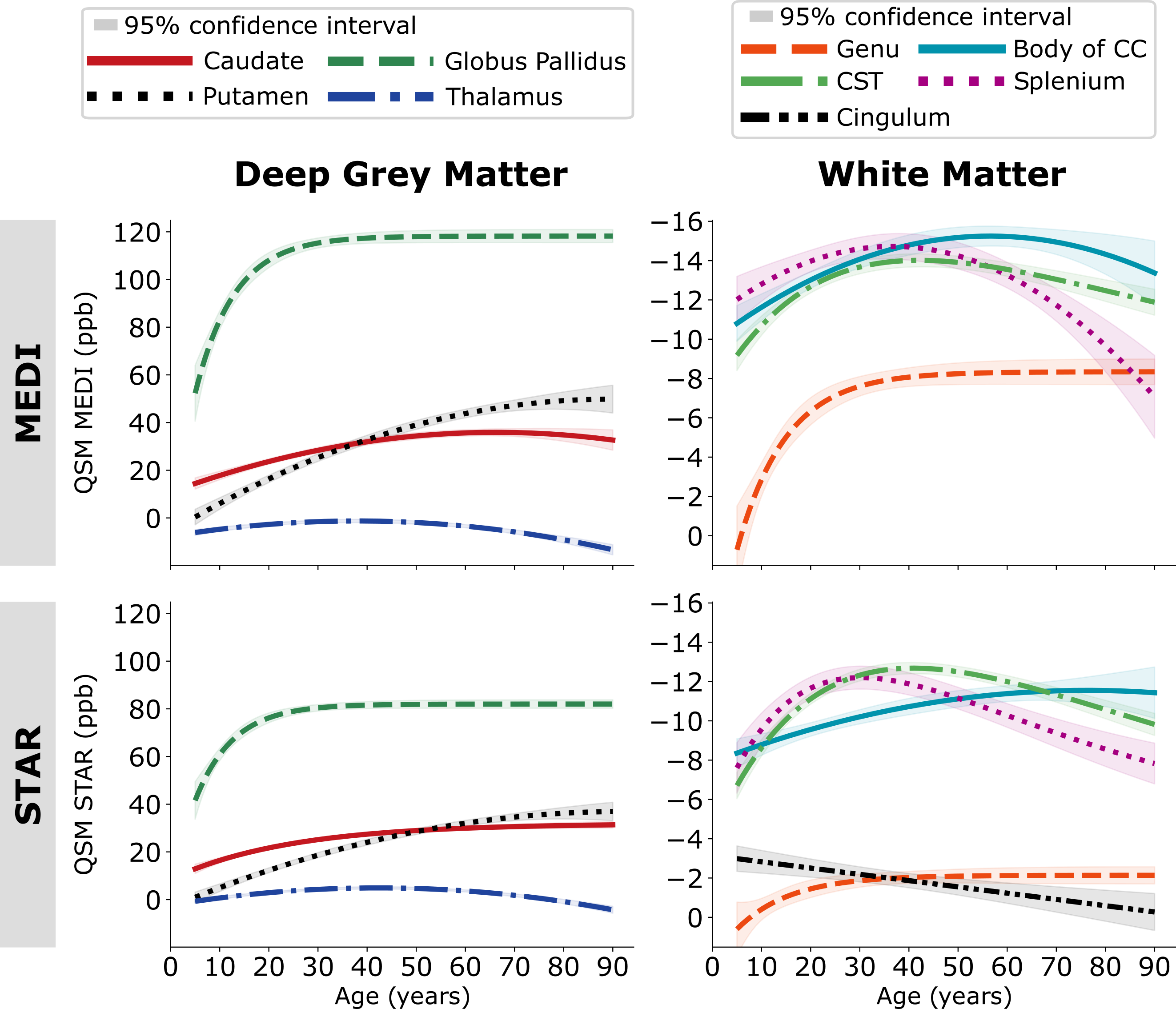


**Supplementary Figure 8.** Best-fit curves for QSM in deep grey matter (left column) and white matter (right column) regions, with MEDI (top row) and STAR (bottom row). Shaded areas show 95% confidence interval, color-coded to each curve. Cingulum QSM MEDI best-fit was not significant, so no curve shown. CC, Corpus callosum; CST, Corticospinal tract.


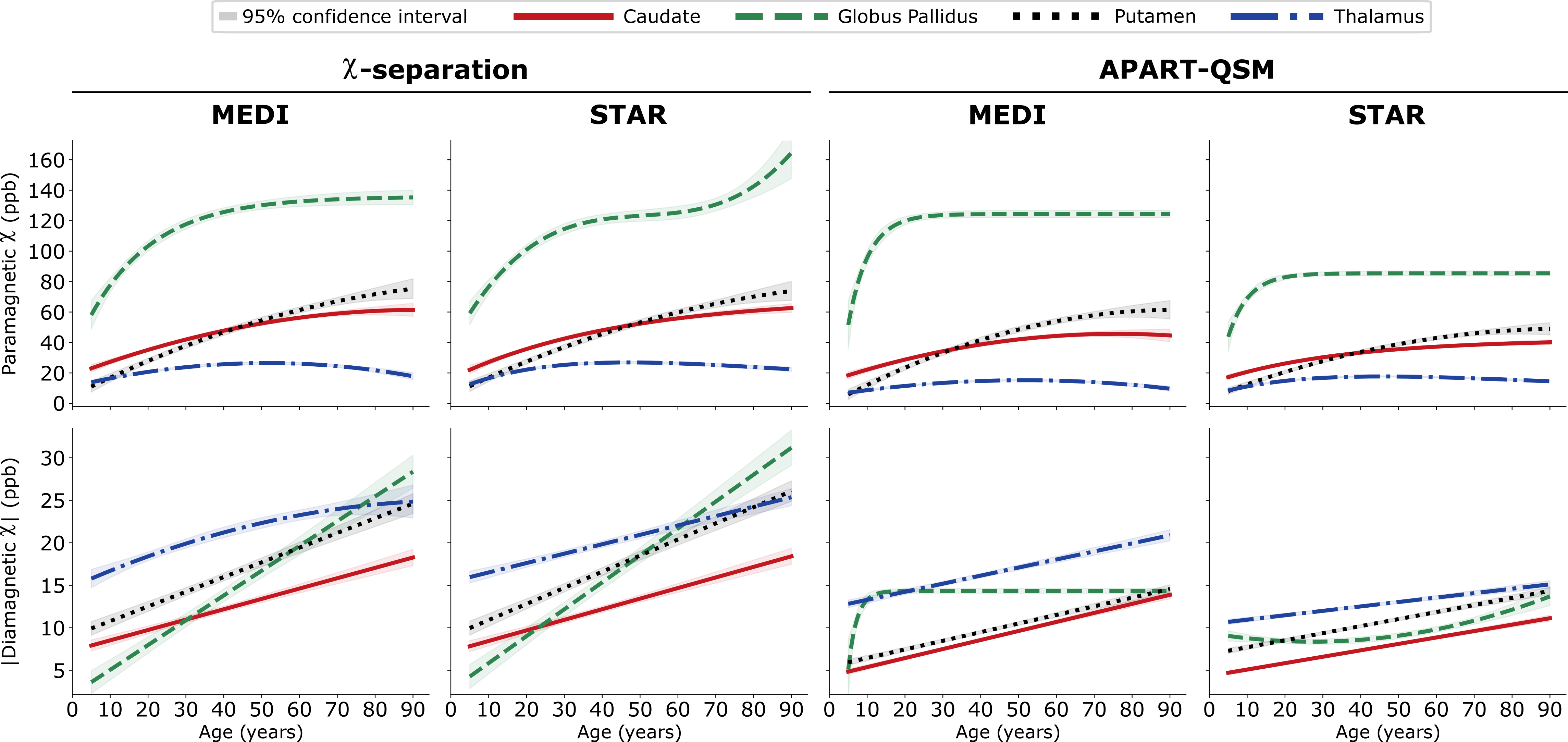


**Supplementary Figure 9.** Effect of QSM method on deep grey matter best fit curves of paramagnetic χ and absolute diamagnetic χ maps, from χ-separation and APART-QSM with MEDI or STAR QSM as first guess inputs. Shaded areas show 95% confidence interval, color-coded to each curve. The paramagnetic χ and absolute diamagnetic χ curves from χ-separation with MEDI and from APART-QSM with STAR are also shown in Figure 4.


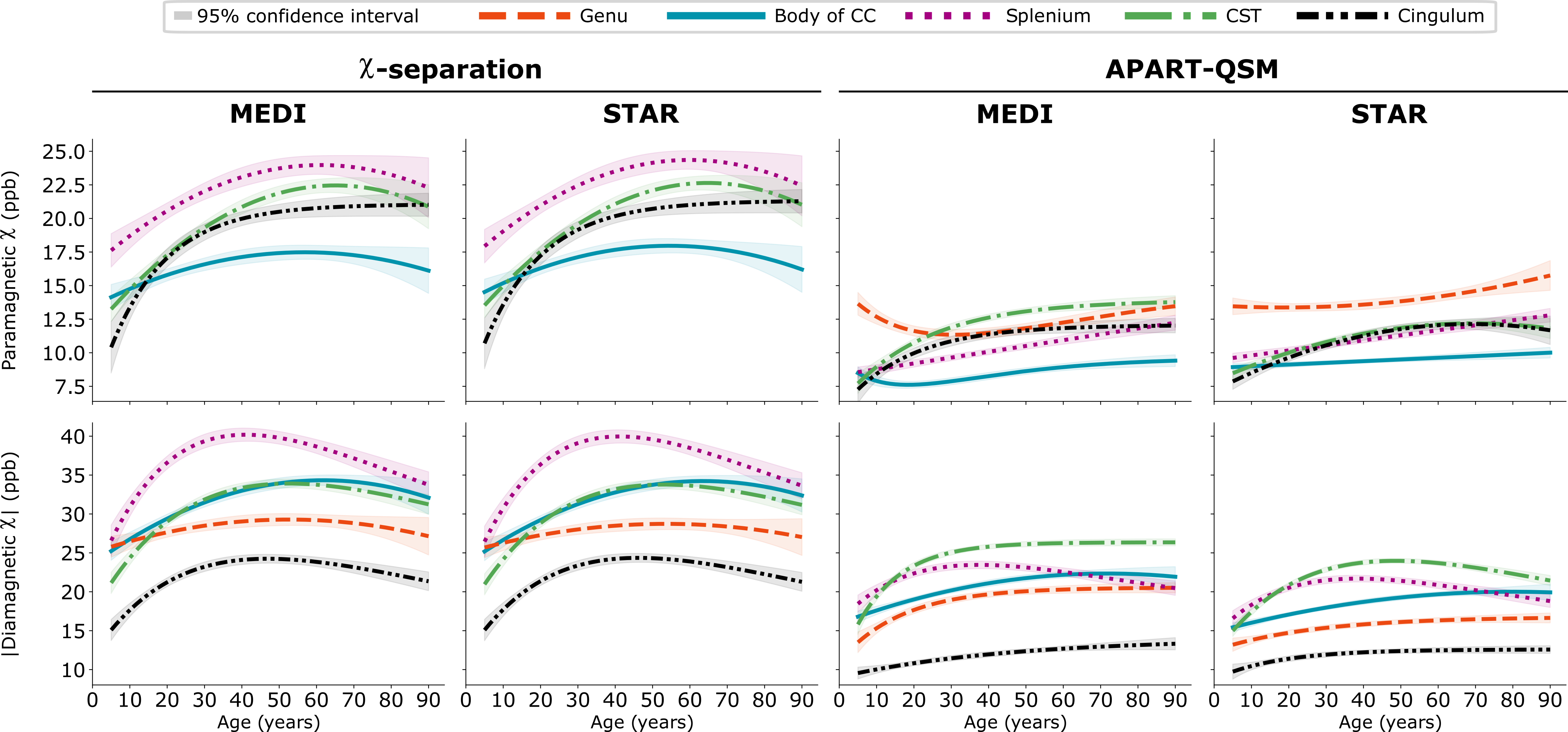


**Supplementary Figure 10.** Effect of QSM method on white matter best-fit curves of paramagnetic χ and absolute diamagnetic χ maps, from χ-separation and APART-QSM with MEDI and STAR QSM as first guess inputs. Shaded areas show 95% confidence interval, color-coded to each curve. Genu paramagnetic χ best-fits with χ-separation (independent of first guess QSM) were not significant, so no curve shown. The paramagnetic χ and absolute diamagnetic χ curves from χ-separation with MEDI and from APART-QSM with STAR are also shown in Figure 5. CC, Corpus callosum; CST, Corticospinal tract.


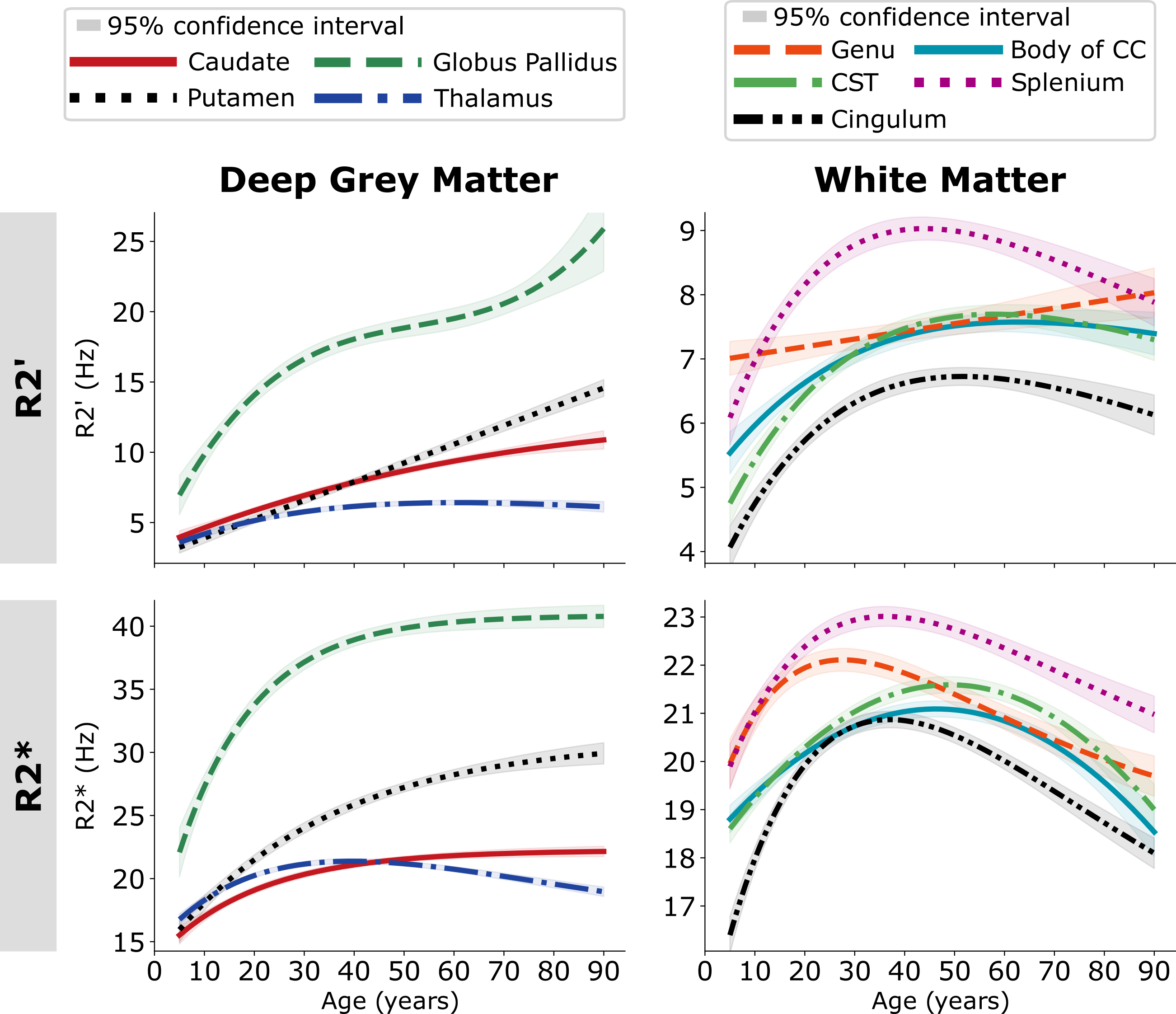


**Supplementary Figure 11.** Best-fit curves in deep grey matter (left column) and white matter (right column) regions for relaxation maps: R2’ (top row) and R2* (bottom row). Shaded areas show 95% confidence interval, color-coded to each curve. CC, Corpus callosum; CST, Corticospinal tract.

**TABLES**

**Supplementary Table 1.** Linear correlation between susceptibility source separation methods. Values shown are Pearson correlation coefficients for paramagnetic and diamagnetic χ for all ROIs and subjects. Values below 0.60 are in bold.

|  |  | **χ-separation vs.**  **χ-sepnet** | **χ-separation vs. APART-QSM** | **χ-sepnet vs.**  **APART-QSM** |
| --- | --- | --- | --- | --- |
| **Deep grey matter** | **Caudate** |  |  |  |
|  | Paramagnetic χ | 0.96 | 0.94 | 0.96 |
|  | Diamagnetic χ | 0.67 | 0.89 | **0.55** |
|  | **Globus pallidus** |  |  |  |
|  | Paramagnetic χ | 0.96 | 0.88 | 0.91 |
|  | Diamagnetic χ | 0.89 | 0.69 | 0.78 |
|  | **Putamen** |  |  |  |
|  | Paramagnetic χ | 0.98 | 0.98 | 0.99 |
|  | Diamagnetic χ | **0.40** | 0.89 | **0.30** |
|  | **Thalamus** |  |  |  |
|  | Paramagnetic χ | 0.87 | 0.90 | 0.92 |
|  | Diamagnetic χ | 0.71 | 0.87 | 0.75 |
|  |  |  |  |  |
| **White matter** | **Genu** |  |  |  |
|  | Paramagnetic χ | 0.72 | 0.74 | 0.68 |
|  | Diamagnetic χ | 0.77 | 0.82 | 0.68 |
|  | **Body of CC** |  |  |  |
|  | Paramagnetic χ | **0.59** | 0.88 | 0.72 |
|  | Diamagnetic χ | 0.69 | 0.89 | 0.70 |
|  | **Splenium** |  |  |  |
|  | Paramagnetic χ | 0.74 | 0.91 | 0.83 |
|  | Diamagnetic χ | 0.77 | 0.87 | 0.77 |
|  | **CST** |  |  |  |
|  | Paramagnetic χ | 0.82 | 0.90 | 0.89 |
|  | Diamagnetic χ | 0.76 | 0.87 | 0.88 |
|  | **Cingulum** |  |  |  |
|  | Paramagnetic χ | 0.76 | 0.92 | 0.81 |
|  | Diamagnetic χ | 0.66 | 0.85 | 0.75 |
|  |  |  |  |  |

*Abbreviations:* CC, Corpus callosum; CST, Corticospinal tract.

**Supplementary Table 2.** QSM peak values and changes from 5 to 90 years from best-fit curves.

|  |  | **Value at 5 years (ppb)** | **Age at peak value (years)** | **Value at 90 years (ppb)** | **Age at 90% of total change from 5 years to peak (years)^a^** | **% change from 5 to 20 years (%)** |
| --- | --- | --- | --- | --- | --- | --- |
| **QSM** | **Caudate** |  |  |  |  |  |
|  | MEDI | 14.5 | 66 | 32.7 | 47 | 43.0 |
|  | STAR | 12.9 |  | 31.4 | 55 | 47.6 |
|  | **Globus pallidus** |  |  |  |  |  |
|  | MEDI | 52.3 |  | 118.2 | 23 | 84.6 |
|  | STAR | 41.6 |  | 82.0 | 23 | 85.7 |
|  | **Putamen** |  |  |  |  |  |
|  | MEDI | 0.4 |  | 49.8 | 63 | 32.3 |
|  | STAR | 1.0 |  | 36.9 | 65 | 31.4 |
|  | **Thalamus** |  |  |  |  |  |
|  | MEDI | -6.1 | 38 | -13.3 | 28 | 70.3 |
|  | STAR | -0.7 | 42 | -4.3 | 31 | 64.3 |
|  | **Genu** |  |  |  |  |  |
|  | MEDI | 0.7 |  | -8.3 | 28 | 77.8 |
|  | STAR | 0.6 |  | -2.1 | 30 | 74.8 |
|  | **Body** |  |  |  |  |  |
|  | MEDI | -10.8 | 57 | -13.4 | 40 | 49.8 |
|  | STAR | -8.4 | 76 | -11.4 | 54 | 37.7 |
|  | **Splenium** |  |  |  |  |  |
|  | MEDI | -12.0 | 37 | -7.1 | 27 | 72.3 |
|  | STAR | -7.6 | 30 | -7.8 | 21 | 88.4 |
|  | **CST** |  |  |  |  |  |
|  | MEDI | -9.2 | 42 | -11.9 | 28 | 72.4 |
|  | STAR | -6.7 | 41 | -9.8 | 27 | 73.9 |
|  | **Cingulum** |  |  |  |  |  |
|  | MEDI^b^ |  |  |  |  |  |
|  | STAR | -3.0 |  | -0.3 | 82 | 17.6 |

*Note:* ^a^For fits that do not have a peak, the 90% of total change was calculated between 5 to 90 years old. ^b^Cingulum MEDI QSM best fit was not significant.

*Abbreviations:* CST, Corticospinal tract.

**Supplementary Table 3.** Parameter values for best-fit curves for QSM.

|  |  | **Fit** | **A** | **B** | **C** | **D** | **E** |
| --- | --- | --- | --- | --- | --- | --- | --- |
| **QSM** | **Caudate** |  |  |  |  |  |  |
|  | MEDI | Quadratic | 10.81 | 0.76 | -5.72E-03 |  |  |
|  | STAR | Exponential | 31.92 |  |  | -23.38 | -0.04 |
|  | **Globus pallidus** |  |  |  |  |  |  |
|  | MEDI | Exponential | 118.22 |  |  | -123.00 | -0.12 |
|  | STAR | Exponential | 82.01 |  |  | -77.30 | -0.13 |
|  | **Putamen** |  |  |  |  |  |  |
|  | MEDI | Quadratic | -5.64 | 1.24 | -6.91E-03 |  |  |
|  | STAR | Quadratic | -3.26 | 0.87 | -4.72E-03 |  |  |
|  | **Thalamus** |  |  |  |  |  |  |
|  | MEDI | Quadratic | -7.68 | 0.34 | -4.46E-03 |  |  |
|  | STAR | Quadratic | -2.31 | 0.34 | -4.03E-03 |  |  |
|  | **Genu** |  |  |  |  |  |  |
|  | MEDI | Exponential | -8.34 |  |  | 14.96 | -0.10 |
|  | STAR | Exponential | -2.14 |  |  | 4.34 | -0.09 |
|  | **Body** |  |  |  |  |  |  |
|  | MEDI | Quadratic | -9.90 | -0.19 | 1.67E-03 |  |  |
|  | STAR | Quadratic | -7.89 | -0.10 | 6.30E-04 |  |  |
|  | **Splenium** |  |  |  |  |  |  |
|  | MEDI | Quadratic | -11.10 | -0.20 | 2.69E-03 |  |  |
|  | STAR | Poisson | -4.77 |  |  | -0.67 | -0.03 |
|  | **CST** |  |  |  |  |  |  |
|  | MEDI | Poisson | -7.24 |  |  | -0.44 | -0.02 |
|  | STAR | Poisson | -4.21 |  |  | -0.56 | -0.02 |
|  | **Cingulum** |  |  |  |  |  |  |
|  | MEDI^a^ |  |  |  |  |  |  |
|  | STAR | Linear | -3.14 | 0.03 |  |  |  |

*Note: Fits were modeled based on the A-E parameters as follows: Linear =* $A + B\cdot age$*; Quadratic =* $A + B\cdot age + C\cdot{age}^{2}$*; Exponential =* $A+D\cdot e^{E\cdot age}$*; Poisson =* $A+D\cdot age\cdot e^{E\cdot age}$*.* ^a^Cingulum MEDI QSM best fit was not significant.

*Abbreviations:* CST, Corticospinal tract.

**Supplementary Table 4.** R2’ and R2* peak values and changes from 5 to 90 years from best-fit curves.

|  |  | **Value at 5 years (ppb)** | **Age at peak value (years)** | **Value at 90 years (ppb)** | **Age at 90% of total change from 5 years to peak (years)^a^** | **% change from 5 to 20 years (%)** |
| --- | --- | --- | --- | --- | --- | --- |
| **Relaxation maps** | **Caudate** |  |  |  |  |  |
|  | R2’ | 4.0 |  | 10.9 | 74 | 27.5 |
|  | R2* | 15.5 |  | 22.1 | 48 | 53.8 |
|  | **Globus pallidus** |  |  |  |  |  |
|  | R2’ | 6.9 |  | 25.9 | 85 | 37.5 |
|  | R2* | 22.1 |  | 40.8 | 40 | 62.5 |
|  | **Putamen** |  |  |  |  |  |
|  | R2’ | 3.2 |  | 14.6 | 82 | 17.6 |
|  | R2* | 15.9 |  | 29.9 | 64 | 39.7 |
|  | **Thalamus** |  |  |  |  |  |
|  | R2’ | 3.6 | 61 | 6.1 | 39 | 55.0 |
|  | R2* | 16.8 | 40 | 19.0 | 26 | 75.5 |
|  | **Genu** |  |  |  |  |  |
|  | R2’ | 7.0 |  | 8.0 | 81 | 17.6 |
|  | R2* | 20.0 | 28 | 19.7 | 19 | 92.3 |
|  | **Body** |  |  |  |  |  |
|  | R2’ | 5.5 | 63 | 7.4 | 41 | 53.7 |
|  | R2* | 18.8 | 46 | 18.5 | 33 | 59.4 |
|  | **Splenium** |  |  |  |  |  |
|  | R2’ | 6.1 | 44 | 7.9 | 29 | 70.3 |
|  | R2* | 19.9 | 36 | 21.0 | 24 | 79.9 |
|  | **CST** |  |  |  |  |  |
|  | R2’ | 4.8 | 58 | 7.3 | 38 | 57.2 |
|  | R2* | 18.6 | 49 | 19.0 | 35 | 56.5 |
|  | **Cingulum** |  |  |  |  |  |
|  | R2’ | 4.1 | 52 | 6.1 | 34 | 62.6 |
|  | R2* | 16.4 | 37 | 18.1 | 25 | 78.9 |

*Note:* ^a^For fits that do not have a peak, the 90% of total change was calculated between 5 to 90 years old.

*Abbreviations:* CST, Corticospinal tract.

**Supplementary Table 5.** Parameter values for best-fit curves for relaxation maps (R2’ and R2*).

|  |  | **Fit** | **A** | **B** | **C** | **D** | **E** |
| --- | --- | --- | --- | --- | --- | --- | --- |
| **Relaxation maps** | **Caudate** |  |  |  |  |  |  |
|  | R2’ | Poisson | 3.24 |  |  | 0.15 | -0.01 |
|  | R2* | Exponential | 22.24 |  |  | -8.65 | -0.05 |
|  | **Globus pallidus** |  |  |  |  |  |  |
|  | R2’ | Cubic | 3.45 | 0.76 | -1.00E-02 |  |  |
|  | R2* | Exponential | 40.85 |  |  | -25.98 | -0.07 |
|  | **Putamen** |  |  |  |  |  |  |
|  | R2’ | Linear | 2.57 | 0.13 |  |  |  |
|  | R2* | Exponential | 31.05 |  |  | -17.59 | -0.03 |
|  | **Thalamus** |  |  |  |  |  |  |
|  | R2’ | Poisson | 2.84 |  |  | 0.16 | -0.02 |
|  | R2* | Poisson | 14.76 |  |  | 0.46 | -0.03 |
|  | **Genu** |  |  |  |  |  |  |
|  | R2’ | Linear | 6.95 | 0.01 |  |  |  |
|  | R2* | Poisson | 18.45 |  |  | 0.36 | -0.04 |
|  | **Body** |  |  |  |  |  |  |
|  | R2’ | Poisson | 5.03 |  |  | 0.11 | -0.02 |
|  | R2* | Quadratic | 18.22 | 0.12 | -1.34E-03 |  |  |
|  | **Splenium** |  |  |  |  |  |  |
|  | R2’ | Poisson | 4.96 |  |  | 0.25 | -0.02 |
|  | R2* | Poisson | 18.38 |  |  | 0.35 | -0.03 |
|  | **CST** |  |  |  |  |  |  |
|  | R2’ | Poisson | 3.94 |  |  | 0.18 | -0.02 |
|  | R2* | Quadratic | 17.88 | 0.15 | -1.54E-03 |  |  |
|  | **Cingulum** |  |  |  |  |  |  |
|  | R2’ | Poisson | 3.23 |  |  | 0.18 | -0.02 |
|  | R2* | Poisson | 14.28 |  |  | 0.49 | -0.03 |

*Note: Fits were modeled based on the A-E parameters as follows: Linear =* $A + B\cdot age$*; Quadratic =* $A + B\cdot age + C\cdot{age}^{2}$*; Exponential =* $A+D\cdot e^{E\cdot age}$*; Poisson =* $A+D\cdot age\cdot e^{E\cdot age}$*.*

*Abbreviations:* CST, Corticospinal tract.
